# Convenient alternative synthesis of the *Malassezia*-derived virulence factor malassezione and related compounds

**DOI:** 10.3762/bjoc.21.135

**Published:** 2025-08-28

**Authors:** Karu Ramesh, Stephen L Bearne

**Affiliations:** 1 Department of Biochemistry & Molecular Biology, Dalhousie University, Halifax, Nova Scotia, B3H 4R2, Canada; 2 Department of Chemistry, Dalhousie University, Halifax, Nova Scotia, B3H 4R2, Canada

**Keywords:** bis(aryl/heteroaryl)ketones, EDC, indole alkaloids, malassezione

## Abstract

Lipophilic yeasts of the genus *Malassezia* are commensal fungi that constitute the normal skin microbiota but may become pathogenic. These fungi, especially *M. furfur*, convert tryptophan into various alkaloid indoles such as malassezione, which may serve as virulence factors. To facilitate testing of malassezione as an aryl hydrocarbon receptor agonist and potential glucokinase activator, we developed a convenient synthetic route from commercially available indole-3-acetic acid. Treatment of the *N*-Boc-protected indole-3-acetic acid with *N*-ethyl-*N*′-(3-dimethylaminopropyl)carbodiimide (EDC) in the presence of DMAP generates the *N*,*N*′-Boc-protected malassezione, which upon deprotection yields malassezione in an overall yield of ca. 20%. This is an improvement over the preparation of the isonitrile followed by an Fe hydride initiated isonitrile–olefin intramolecular coupling reaction, which generated malassezione with an overall yield of ca. 5%. Furthermore, the present method may also be used to prepare related compounds.

## Introduction

**Figure 1:**
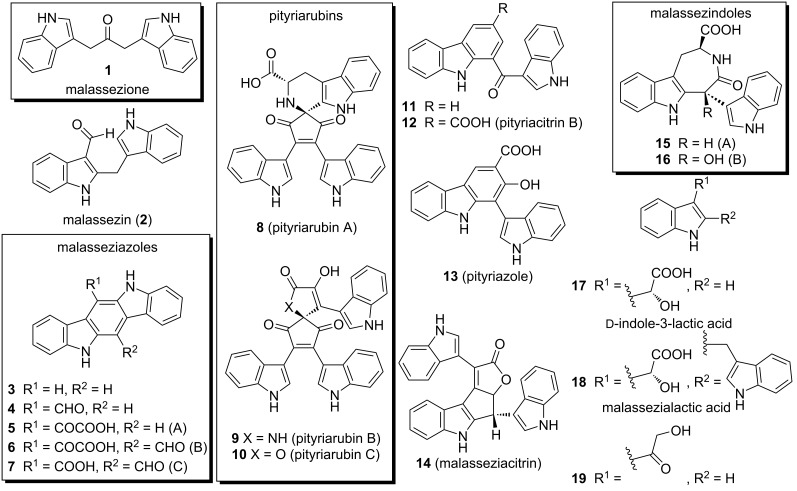
Various natural indole-containing compounds isolated from *Malassezia furfur*.

Lipophilic yeasts of the genus *Malassezia* are commensal fungi that constitute the normal skin microbiota; however, when they become pathogenic, they are typically associated with various skin diseases, including pityriasis versicolor, malassezia folliculitis, atopic dermatitis, and seborrhoeic dermatitis in humans [1–7]. Additionally, *Malassezia* DNA has been suggested to play a role in the etiology in neurological disorders [7] and possibly other non-skin diseases [5]. These fungi, especially *M. furfur*, convert tryptophan into various alkaloid indoles such as malassezione (**1**), malassezin (**2**), which cyclizes to indolo[3,2-*b*]carbazole (**3**), other related indolo[3,2-*b*]carbazoles (**4**–**7**), pityriarubins (**8**–**10**), and others (**11**–**19**) (see Figure 1), which may serve as virulence factors [8–13]. Most significantly, some of these indoles have been shown to be potent aryl hydrocarbon receptor (AHR) ligands [8–9,14–15], which can lead to induction of melanocyte apoptosis and inhibition of neutrophil function [16–17].

Malassezione (**1**, also referred to as malathidone [18]) is an AHR agonist [19–20]. Recently, in an effort to identify compounds as glucokinase activators to treat type 2 diabetes, structure-based virtual screening identified malassezione as a potential glucokinase activator [21]. Unfortunately, testing of malassezione is limited by the lack of a convenient synthesis. Indeed, beyond isolating the compound from cultured *M. furfur* [11,20], the only reported synthetic route to malassezione is via an iron-catalyzed Fukuyama-type indole synthesis, which afforded the compound in low yield [18]. Herein, we report an alternative synthesis from commercially available indole acetic acid, and demonstrate that the approach can be extended to related compounds.

## Results and Discussion

**Scheme 1:**
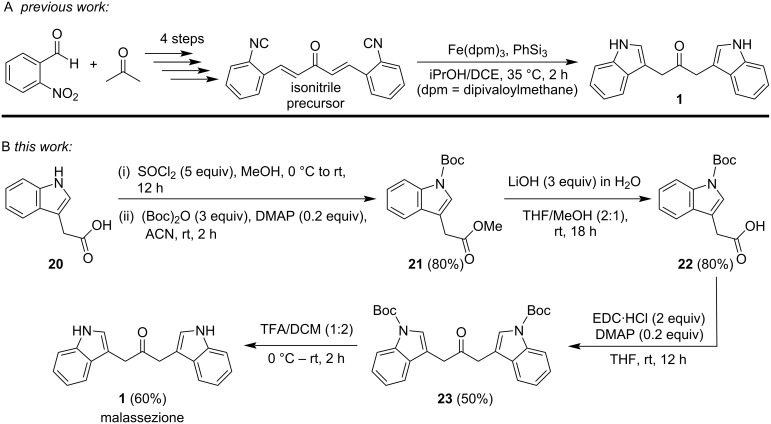
Synthetic routes to malassezione (**1**) from either A) an isonitrile precursor [18] or B) indole-3-acetic acid.

Previously, malassezione had either been (i) isolated from cultures of *M. furfur* grown on ʟ-tryptophan as the sole nitrogen source followed by thin-layer chromatography and reversed-phase (RP)-HPLC to isolate and purify the compound from a complex mixture of indole alkaloids [11], (ii) prepared by aerobic incubation of indole-3-pyruvic acid at pH 7.4, 37 °C for 24 h followed by isolation and purification using RP-HPLC [20], or, more recently, (iii) prepared via an Fe hydride initiated isonitrile–olefin intramolecular coupling reaction (Scheme 1A) [18]. The reported synthetic route relies on an initial 4-step preparation of the isonitrile precursor, which was accomplished in an overall yield of ca. 14%. The subsequent iron-catalyzed reaction afforded **1** in only 35% yield. To avoid the low-yielding preparation of the isonitrile, we elected to use commercially available indole-3-acetic acid as the starting material, and to utilize an approach similar to that described for the synthesis of dibenzylketones from the DCC-induced condensation of phenylacetic acid reported by Bhandari and Ray [22] and others [23–27]. Initially, we converted indole-3-acetic acid to its methyl ester, followed by protection of the indole nitrogen with a Boc group and subsequent hydrolysis of the methyl ester to regenerate the carboxylic acid (Scheme 1B) in a manner similar to that described by Knör et al. [28]. Protection of the carboxylic acid group prior to conducting the *N*-Boc protection with di-*tert*-butyl dicarbonate ((Boc)_2_O) and DMAP was required to avoid competitive reaction of the carboxylic acid with (Boc)_2_O to form a *tert*-butyl ester, leading to unwanted side products and reduced selectivity. Once the acid was protected, the Boc group could be selectively introduced onto the indole nitrogen without side reactions. Overall, this sequence ensured clean *N*-Boc protection with minimal side reactions and easier purification. The *N*-Boc-protected indole-3-acetic acid was subsequently treated with *N*-ethyl-*N*′-(3-dimethylaminopropyl)carbodiimide (EDC) [29] in the presence of DMAP to generate the *N*,*N*′-Boc-protected malassezione (**23**). Use of EDC greatly facilitated the purification since the resulting urea derivative was easily removed with aqueous washes, thereby obviating the need for column purification to remove the more hydrophobic dicyclohexylurea obtained when DCC was employed to facilitate the reaction. Subsequent removal of the Boc groups with TFA furnished malassezione (**1**) in an overall yield of ca. 20% (4 steps) relative to an overall yield of ca. 5% via the preparation of the isonitrile followed by the Fe hydride initiated isonitrile–olefin intramolecular coupling reaction [18]. Protection of the indole nitrogens with the Boc group was preferable to the use of benzyl protecting groups since removal of the benzyl groups by hydrogenation was problematic (vide infra*)*. The NMR spectral characteristics of the resulting material were identical to those reported for malassezione either isolated from cultures [11,20] or prepared from the isonitrile [18].

**Scheme 2:**
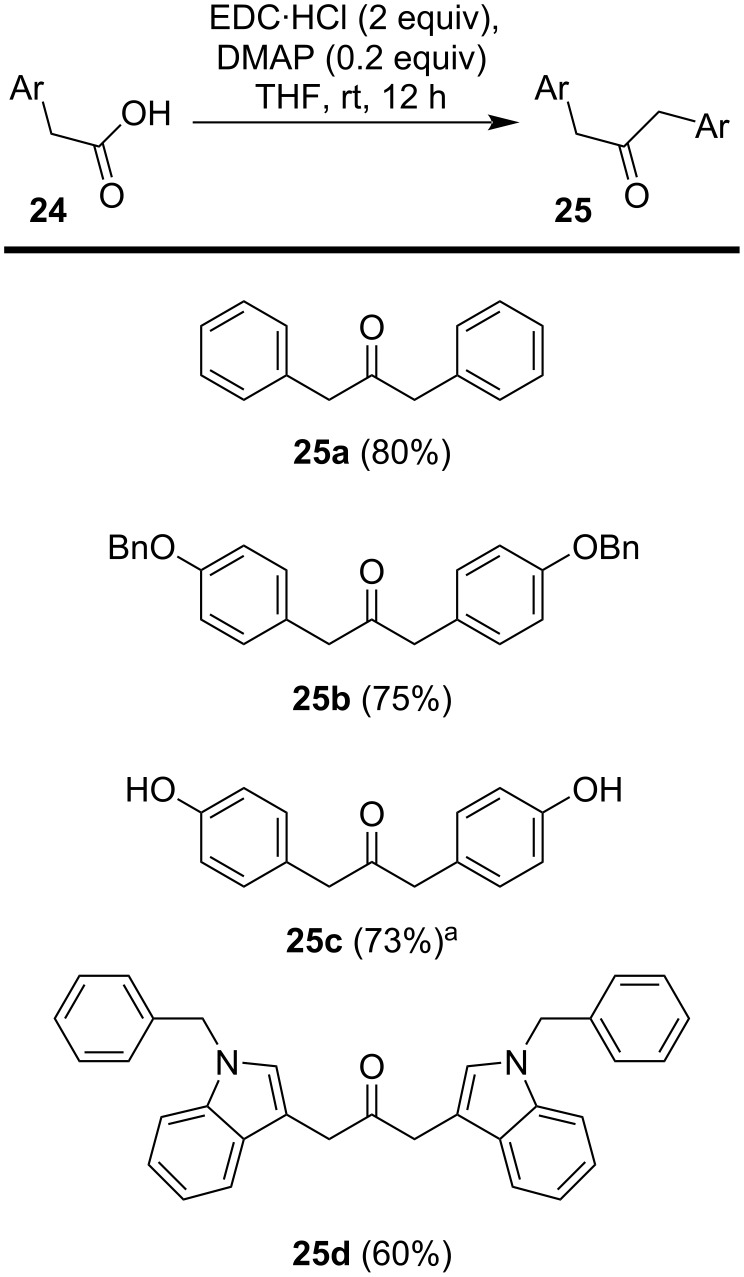
Various bis-substituted ketones prepared. ^a^**25c** prepared from **25b**.

To further demonstrate the utility of the approach, we also prepared the dibenzylketone (**25a**) from phenylacetic acid in 80% yield (cf. 64% yield reported by Bhandari and Ray [22]) (Scheme 2). Previously, Bhandari and Ray demonstrated that the DCC-mediated reaction could be used with 4-methoxyphenylacetic acid to generate bis(4-methoxybenzyl) ketone, which could be converted to the corresponding bis(4-hydroxybenzyl) ketone (**25c**) by heating at 200 °C for 15 min to 3 h in the presence of pyridinium chloride [22,24]. Herein, we also show that the EDC-mediated coupling of 4-(benzyloxy)phenylacetic acid readily yields **25b**, which may be debenzylated by Pd-catalyzed hydogenation to cleanly yield **25c**. This affords a route to such compounds bearing hydroxylated aromatic groups that employs milder conditions than previously reported [22,24]. Finally, we prepared *N*,*N*′-dibenzylmalassezione (**25d**). Initially, this compound was prepared with the intent of using the benzyl group to protect the indole nitrogen rather than the Boc group. However, Pd-catalyzed hydrogenation of **25d** led to a mixture of products, of which some were consistent with reduction of the indole ring.

With respect to the scalability of the synthesis, the reactions can be conducted on the gram-scale starting from indole-3-acetic acid with consistently good yields up to and including the EDC-mediated coupling step. However, the subsequent removal of the Boc protecting group proved more challenging. We observed lower yields during this step, despite screening a range of acids and conditions, including variations of the TFA treatment. Among the conditions tested, TFA gave the best results, though an optimal outcome was achieved only on the smaller, 100 mg scale. Scaling up the reaction at this stage increased the formation of side products. Consequently, it might be advisable to stockpile the penultimate synthetic intermediate **23** and subsequently conduct the deprotection on the smaller scale.

## Conclusion

Overall, we have developed a facile synthesis of the natural product malassezione, utilizing an EDC-mediated coupling of *N*-Boc-protected indole-3-acetic acid. The use of EDC permits easy removal of the water-soluble urea-based byproduct with an aqueous wash as opposed to the hydrophobic dicyclohexylurea arising when DCC is utilized, which is more difficult to separate from the hydrophobic malassezione. This route should afford ready access to malassezione for assessing its role as an AHR agonist [19–20] and potential glucokinase activator [21].

## Experimental

**General.** Unless otherwise noted, all reagents were used as received from commercial sources: Sigma-Aldrich Canada Ltd. (Oakville, ON, Canada), TCI America (Portland, OR), or Fisher Scientific Canada (Ottawa, ON, Canada). Reactions were monitored using thin-layer chromatography on aluminium-backed silica plates (Sigma-Aldrich) using either UV-light (254 nm), iodine, KMnO_4_, phosphomolybdic acid, or *p*-anisaldehyde for visualization. Tetrahydrofuran (THF) was dried and distilled over sodium/benzophenone. Concentration under reduced pressure refers to the removal of solvent using a rotary evaporator, unless stated otherwise. All NMR spectra were obtained using a Bruker ASCEND 400 MHz spectrometer at the Nuclear Magnetic Resonance Research Resource (NMR-3, Dalhousie University). Chemical shifts (δ in ppm) for proton (^1^H) spectra are reported relative to the residual solvent signal for CDCl_3_ (δ 7.26) or methanol-*d*_4_ (δ 3.31) [30]. Chemical shifts (δ in ppm) for carbon (^13^C) spectra are reported relative to the residual solvent signal for CDCl_3_ (δ 77.16) or methanol-*d*_4_ (δ 49.00) [30]. Abbreviations used for reporting NMR spectral data are: bm, broad multiplet; bs, broad singlet; bt, broad triplet; d, doublet; dd, doublet of doublets; m, multiplet; q, quartet; s, singlet; and t, triplet. High-resolution (HR) electrospray ionization (ESI) mass spectra (MS) were collected using a Bruker microTOF Focus orthogonal ESI-TOF mass spectrometer instrument operating in either negative or positive ion mode as indicated in the experimental procedures (Chemistry Mass Spectrometry Core Facility, Dalhousie University). Melting points were recorded on Barnstead International (Model No. 1201D) melting point apparatus and are uncorrected.

***tert*-Butyl 3-(2-methoxy-2-oxoethyl)-1*H*-indole-1-carboxylate (21).** A solution of indol-3-yl acetic acid (**20**, 5.1 g, 29.0 mmol, 1.0 equiv) in dry MeOH (200 mL) was cooled to 0 °C, and SOCl_2_ (10.5 mL, 145 mmol, 5.0 equiv) was added slowly. After stirring for 12 h at room temperature, the solution was concentrated under reduced pressure. The residue was taken up in EtOAc (200 mL) and subsequently washed with saturated aqueous NH_4_Cl (150 mL), saturated aqueous NaHCO_3_ (150 mL), and brine (150 mL). After drying over anhydrous MgSO_4_, the mixture was concentrated to dryness to give the pure methyl ester as a brown oil (5.1 g, 94%); *R*_f_ = 0.3 (EtOAc/hexane 1:2). Spectroscopic data for the methyl ester agreed with those in the literature [31]. The methyl ester (5.0 g, 26.0 mmol, 1.0 equiv) was then dissolved in acetonitrile (25 mL) and di-*tert*-butyl dicarbonate (17.0 g, 78 mmol, 3.0 equiv) and DMAP (0.63 g, 5.2 mmol, 0.2 equiv) were added [32]. The mixture was stirred for 2 h at room temperature after which the solvent was removed under reduced pressure. The residue was dissolved in EtOAc (250 mL) and subsequently washed with saturated aqueous NH_4_Cl (150 mL), saturated aqueous NaHCO_3_ (150 mL), and brine (100 mL). After drying over anhydrous MgSO_4_, the solvent was removed in vacuo and the crude product was purified by flash chromatography on silica gel (EtOAc/hexane; gradient 1:10 → 1:2) to give **21** as a colorless solid (6.7 g, 80%, 2 steps); *R*_f_ = 0.5 (EtOAc/hexane 1:2); mp 50–52 °C (lit. mp 53 °C [28]); ^1^H NMR (400 MHz, CDCl_3_) δ 8.17 (d, *J =* 8.1 Hz, 1H, ArH), 7.58 (s, 1H, ArH), 7.54 (dt, *J =* 7.7 and 1.1 Hz, 1H, ArH), 7.34 (ddd, *J =* 8.4, 7.2 and 1.1 Hz, 1H, ArH), 7.26 (ddd, *J =* 8.4, 7.2 and 1.1 Hz, 1H, ArH), 3.73 (d, *J =* 1.1 Hz, 2H), 3.71 (s, 3H, CH_3_), 1.67 (s, 9H, 3 × CH_3_) ppm; ^13^C NMR (100 MHz, CDCl_3_) δ 171.5, 149.6, 135.5, 130.1, 124.6, 124.5, 122.7, 119.0, 115.3, 113.1, 83.6, 52.1, 30.9, 28.2 ppm. These spectroscopic data agreed with those in the literature [28].

**2-(1-(*tert*-Butoxycarbonyl)-1*H*-indol-3-yl)acetic acid (22).** A solution of lithium hydroxide (LiOH·H_2_O, 2.1 g, 51.8 mmol, 3 equiv) in H_2_O (250 mL) was added to a solution of **21** (5.0 g, 17.2 mmol, 1.0 equiv) in THF (300 mL) and MeOH (150 mL). After stirring for 18 h at room temperature, the mixture was concentrated in vacuo and a 10% aqueous solution of citric acid (100 mL) was added. The aqueous layer was extracted with EtOAc (3 × 50 mL) and the combined organic layers were washed with water (100 mL) and brine (100 mL), and then dried over anhydrous MgSO_4_. The solvent was removed under reduced pressure and the residue was purified by flash chromatography on silica gel (EtOAc/hexane gradient 1:10 → 1:1, 1% AcOH), yielding **22** as a colorless solid (3.7 g, 80%); R_f_ = 0.6 (MeOH/CHCl_3_ 1:9, 1% AcOH); mp 116–118 °C (lit. mp 117–120 °C [28]); ^1^H NMR (400 MHz, CDCl_3_) δ 8.14 (d, *J =* 8.3 Hz, 1H, ArH), 7.58 (s, 1H, ArH), 7.53 (dt, *J* = 7.8 and 1.1 Hz, 1H, ArH), 7.53 (ddd, *J* = 8.4, 7.2 and 1.3 Hz, 1H, ArH), 7.33 (ddd, *J* = 8.4, 7.2 and 1.3 Hz, 1H, ArH), 3.76 (s, *J =* 1.0 Hz, 2H), 1.66 (s, 9H, 3 × CH_3_) ppm; ^13^C NMR (100 MHz, CDCl_3_) δ 176.8, 149.7, 135.5, 135.0, 124.84, 124.80, 122.8, 119.1, 115.5, 112.5, 83.9, 30.9, 28.3 ppm; HRESIMS (*m*/*z*): [M + Na]^+^ calcd for C_15_H_17_NNaO_4_, 298.1050; found, 298.1057. These spectroscopic data agreed with those in the literature [28].

**Di-*tert*-butyl 3,3'-(2-oxopropane-1,3-diyl)bis(1*H*-indole-1-carboxylate) (23).** An oven-dried 50 mL flask equipped with a magnetic stir bar was charged with the Boc-protected acid **22** (1 g, 3.6 mmol, 1 equiv), EDC·HCl (1.3 g, 7.2 mmol, 2 equiv), and DMAP (87 mg, 0.7mmol, 0.2 equiv) in THF (10 mL) under an argon atmosphere. The reaction mixture was stirred at room temperature for 12 h. After completion of the reaction, the solvent was removed under reduced pressure. The crude reaction mixture was then diluted with EtOAc (100 mL) and washed with water (2 × 40 mL), followed by brine (40 mL). The organic layer was dried over anhydrous Na_2_SO_4_, and the solvent was evaporated under reduced pressure. The residue was purified by flash chromatography on silica gel using a gradient of EtOAc/hexane (1:10 → 1:1), affording compound **23** as a colorless viscous oil (425 mg, 50%); ^1^H NMR (400 MHz, CDCl_3_) δ 8.12 (d, *J* = 8.3 Hz, 1H, ArH), 7.49 (s, 1H, ArH), 7.36–7.29 (m, 2H, ArH), 7.20 (td, *J* = 7.5 and 1.0 Hz, 1H, ArH), 3.85 (d, *J* = 1.0 Hz, 2H, CH_2_), 1.66 (s, 9H, 3 × CH_3_) ppm; ^13^C NMR (100 MHz, CDCl_3_) δ 205.0, 149.7, 135.5, 130.2, 124.8, 124.7, 122.8, 119.0, 115.4, 113.2, 83.8, 38.7, 28.3 ppm; HRESIMS (*m*/*z*): [M + Na]^+^ calcd for C_29_H_32_N_2_NaO_5_, 511.2203; found, 511.2222.

**1,3-Di(1*H*-indol-3-yl)propan-2-one (1):** The ketone **23** (200 mg, 0.4 mmol, 1 equiv) was dissolved in DCM (6 mL) in a 25 mL round bottom flask equipped with a magnetic stirrer. Trifluoroacetic acid (3 mL) was then added dropwise at 0 °C. The reaction was then stirred for 2 h as the temperature rose from 0 °C to room temperature. The solution was then concentrated in vacuo to yield an oily residue that was co-evaporated with diethyl ether (3 × 10 mL) under vacuum to produce the desired compound **1** as a beige viscous oil (70 mg, 60%); ^1^H NMR (400 MHz, CDCl_3_) δ 8.07 (s, 1H, NH), 7.47 (dd, *J* = 8.0 and 1.1 Hz, 1H, ArH(4/4′)), 7.35 (dt, *J* = 8.2 and 0.9 Hz, 1H, ArH(7/7′)), 7.20 (ddd, *J* = 8.2, 7.0 and 1.2 Hz, 1H, ArH(6/6′)), 7.11 (ddd, *J* = 8.0, 7.0 and 1.0 Hz, 1H, ArH(5/5′)), 7.05 (d, *J* = 2.4 Hz, 1H, ArH(2/2′)), 3.89 (s, 2H, CH_2_(8/8′)) ppm; ^13^C NMR (100 MHz, CDCl_3_) δ 207.2 (C9/9′), 136.3 (C7a/7a′), 127.5 (C3a/3a′), 123.4 (C2/2′), 122.4 (C6/6′), 119.9 (C5/5′), 118.9 (C4/4′), 113.4 (C7/7′), 108.9 (C3/3′), 38.7 (C8/8′) ppm; HRESIMS (*m*/*z*): [M + Na]^+^ calcd for C_19_H_16_N_2_NaO, 311.1155; found, 311.1163. These data are fully consistent with those published previously [20].


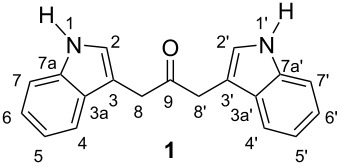


The observation of 19 carbon atoms in **1** (HRESIMS) and 10 carbon signals in the ^13^C NMR spectrum is in accord with the molecule being symmetric. The structure of di-indole **1** was further supported by 2D NMR. The ^1^H-^1^H COSY spectrum showed couplings between the H4-H5, H5-H6, H6-H7, and H1-H2 protons, as well as long-range coupling between the H2-H8 protons. Again, these data are fully consistent with those published previously [20]. Together, these results indicate the presence of two indoles, one carbonyl, one methylene, and one methine group where the connectivities of these groups were supported from the ^1^H-^13^C HMBC spectrum. Couplings between the H8 and C9 (or H8′ and C9) atoms are consistent with the C8 and C8′ atoms linked to the carbonyl carbon (C9). In addition, couplings between the H8 and the C2, C3, C3a, and C8 carbon atoms, as well as between the H2 and the C3, C7a, and C8 carbon atoms, were observed. These couplings are consistent with the C8 and C8′ atoms being attached to the C3 and C3′ carbon atoms of the two indole groups, respectively. Furthermore, the ^1^H-^13^C HMBC spectrum showed couplings in the aromatic region consistent with the presence of the indole groups.

**General procedure for the synthesis of ketones 25a,b, and d from acids 24a,b, and d.** Adapting DCC/DMAP protocols reported previously [22–27], an oven-dried 50 mL flask equipped with a magnetic stir bar was charged with the acid (1 equiv, typically 1.0 g), EDC·HCl (2 equiv), and DMAP (0.2 equiv) in THF (10 mL) under an argon atmosphere. The reaction mixture was stirred at room temperature for 12 h. After completion of the reaction, the solvent was removed under reduced pressure, and the crude reaction mixture was diluted with EtOAc (100 mL), then washed with water (2 × 40 mL) and brine (40 mL). The organic layer was dried over anhydrous Na_2_SO_4_, and the solvent was evaporated under reduced pressure. The resulting residue was subsequently purified by flash chromatography on silica gel using a hexane/ethyl acetate mixture as the eluent.

**1,3-Diphenylpropan-2-one (25a).** The reaction was conducted according to the general procedure using commercially available phenylacetic acid (1.0 g, 7.3 mmol, 1 equiv). The crude product was purified by chromatography on silica gel (hexane/EtOAc; 90:10) to afford **25a** as a white solid (1.2 g, 80%); mp 108–110 °C; ^1^H NMR (400 MHz, CDCl_3_) δ 7.32–7.22 (m, 6H, ArH), 7.14–7.12 (m, 4H, ArH), 3.70 (s, 4H, 2 × CH_2_) ppm; ^13^C NMR (100 MHz, CDCl_3_) δ 205.8, 134.1, 129.6, 128.8, 127.2, 49.2 ppm. These spectroscopic data agreed with those in the literature [22,33–34].

**1,3-Bis(4-(benzyloxy)phenyl)propan-2-one (25b).** The reaction was conducted according to the general procedure using the commercially available 4-(benzyloxy)phenylacetic acid (1.0 g, 4.7 mmol, 1 equiv). The crude product was purified by chromatography on silica gel (hexane/EtOAc; 90:10) to afford **25b** as a white solid (1.2 g, 75%); mp 110–112 °C (lit. mp 119 °C [35]); ^1^H NMR (400 MHz, CDCl_3_) δ 7.46–7.32 (m, 10H, ArH), 7.10–7.06 (m, 4H, ArH), 6.96–6.93 (m, 4H, ArH), 5.07 (s, 4H, 2 × OCH_2_), 3.66 (s, 4H, 2 X × CH_2_) ppm; ^13^C NMR (100 MHz, CDCl_3_) δ 206.5, 158.0, 137.1, 130.6, 128.7, 128.1, 127.6, 126.5, 115.2, 70.1, 48.2 ppm; HRESIMS (*m*/*z*): [M + Na]^+^ calcd for C_29_H_26_NaO_3_, 445.1774; found, 445.1777. These spectral data agreed with those in the literature [35].

**1,3-Bis(4-hydroxyphenyl)propan-2-one (25c).** An oven-dried 25 mL flask equipped with a magnetic stir bar was charged with 1,3-bis(4-(benzyloxy)phenyl)propan-2-one (**25b**, 100 mg, 0.2 mmol, 1 equiv) and dissolved in ethanol (3 mL). A catalytic amount of Pd/C was then added and hydrogenolysis was conducted for 4 h with stirring under an H_2_-filled balloon. The reaction was monitored by TLC and, after completion of the reaction, the mixture was filtered through Celite, followed by washing of the remaining Pd/C with ethanol (3 × 5 mL). The solvent was removed by evaporation under reduced pressure and the resulting crude compound was purified by chromatography on silica gel (hexane/EtOAc; 90:10) to afford **25c** as a white solid (41 mg, 73%); mp 154–156 °C (lit. mp 164 °C [22]; 150–152 °C [35]; 157–158 °C [24]); ^1^H NMR (400 MHz, methanol-*d*_4_) δ 6.96–6.91 (m, 4H, ArH), 6.73–6.69 (m, 4H, ArH), 3.63 (s, 4H, 2 × CH_2_) ppm; ^13^C NMR (100 MHz, methanol-*d*_4_) δ 209.2, 156.8, 131.0, 125.8, 115.7, 47.2 ppm; HRESIMS (*m*/*z*): [M + Na]^+^ calcd for C_15_H_14_NaO_3_, 265.0835; found, 265.0842. These spectral data agreed with those in published reports [22,24,35].

**1,3-Bis(1-benzyl-1*H*-indol-3-yl)propan-2-one (25d).** Indole-3-acetic acid (1.0 g, 5.7 mmol) in DMF (10 mL) was cooled to 0 °C, and sodium hydride (273 mg, 11.4 mmol, 2 equiv) was added slowly with stirring. Benzyl bromide (1.9 g, 11.4 mmol, 2 equiv) was then added dropwise, and the reaction mixture was stirred for 12 h, allowing the temperature to gradually rise to room temperature. Upon completion of the reaction, the mixture was washed with cold LiCl solution (10%) and extracted with ethyl acetate (3 × 40 mL). The combined organic extracts were dried over anhydrous Na_2_SO_4_ and concentrated under reduced pressure to afford 2-(1-benzyl-1*H*-indol-3-yl)acetic acid [36]. The resulting product was then used to synthesize ketone **25d** following the general procedure. The crude product was purified by chromatography on silica gel (hexane/EtOAc; 90:10) to afford **25d** as a yellow viscous oil (52 mg, 60%); ^1^H NMR (400 MHz, CDCl_3_) δ 7.46 (d, *J* = 7.9 Hz, 1H, ArH), 7.30–7.22 (m, 4H, ArH), 7.17 (t, *J* = 7.6 Hz, 1H, ArH), 7.09 (dd, *J* = 7.7 and 2.4 Hz, 3H, ArH), 6.99 (s, 1H), 5.24 (s, 4H, 2 × CH_2_), 6.99 (s, 4H, 2 × CH_2_) ppm; ^13^C NMR (100 MHz, CDCl_3_) δ 207.2, 137.6, 136.7, 128.9, 127.8, 127.5, 127.0, 122.1, 119.6, 119.1, 109.9, 108.1, 50.1, 38.6 ppm; HRESIMS (*m*/*z*): [M + Na]^+^ calcd for C_33_H_28_N_2_NaO_3_, 491.2094; found, 491.2090.

## Supporting Information

File 1 Copies of NMR and MS spectra of synthesized compounds.

## Data Availability

All data that supports the findings of this study are available in the published article and/or the supporting information of this article.
