# Supplementary material for: Convenient alternative synthesis of the Malassezia-derived virulence factor malassezione and related compounds
Source: Beilstein J Org Chem. 2025 Aug 28;21:1730–6. doi: 10.3762/bjoc.21.135 (PMC12415918; doi:10.3762/bjoc.21.135)
Supplement: File 1 — Copies of NMR and MS spectra of synthesized compounds. [file Beilstein_J_Org_Chem-21-1730-s001.pdf]

BEILSTEIN JOURNAL OF ORGANIC CHEMISTRY

Supporting Information

for

Convenient alternative synthesis of the *Malassezia*-derived virulence factor malassezione and related compounds

Karu Ramesh and Stephen L. Bearne

Beilstein J. Org. Chem. **2025,** *21,* 1730–1736. doi:10.3762/bjoc.21.135

Copies of NMR and MS spectra of synthesized compounds

License and Terms: This is a supporting information file under the terms of the Creative Commons Attribution License (https://creativecommons.org/licenses/by/4.0). Please note that the reuse, redistribution and reproduction in particular requires that the author(s) and source are credited and that individual graphics may be subject to special legal provisions.
The license is subject to the *Beilstein Journal of Organic Chemistry* terms and conditions: (https://www.beilstein-journals.org/bjoc/terms)

TABLE OF CONTENTS **PAGE**

Figure S1	^{1}H NMR spectrum of *tert*-butyl 3-(2-methoxy-2-oxoethyl)-1*H*-indole-1-carboxylate (**21**) in $CDCl_3$	S4
Figure S2	^{13}C NMR spectrum of *tert*-butyl 3-(2-methoxy-2-oxoethyl)-1*H*-indole-1-carboxylate (**21**) in $CDCl_3$	S5
Figure S3	^{1}H NMR spectrum of 2-(1-(*tert*-butoxycarbonyl)-1*H*-indol-3-yl)-acetic acid (**22**) in $CDCl_3$	S6
Figure S4	^{13}C NMR spectrum of 2-(1-(*tert*-butoxycarbonyl)-1*H*-indol-3-yl)-acetic acid (**22**) in $CDCl_3$	S7
Figure S5	^{1}H NMR spectrum of di-*tert*-butyl 3,3'-(2-oxopropane-1,3-diyl)-bis(1*H*-indole-1-carboxylate) (**23**) in $CDCl_3$	S8
Figure S6	^{13}C NMR spectrum of di-*tert*-butyl 3,3'-(2-oxopropane-1,3-diyl)-bis(1*H*-indole-1-carboxylate) (**23**) in $CDCl_3$	S9
Figure S7	^{1}H NMR spectrum of 1,3-di(1*H*-indol-3-yl)propan-2-one (**1**) in $CDCl_3$	S10
Figure S8	^{13}C NMR spectrum of 1,3-di(1*H*-indol-3-yl)propan-2-one (**1**) in $CDCl_3$	S11
Figure S9	^{1}H-^{1}H-COSY NMR spectrum of 1,3-di(1*H*-indol-3-yl)propan-2-one (**1**) in $CDCl_3$	S12
Figure S10	Expanded ^{1}H-^{1}H-COSY NMR spectrum of 1,3-di(1*H*-indol-3-yl)propan-2-one (**1**)	S13
Figure S11	Expanded aromatic region of the ^{1}H-^{1}H-COSY NMR spectrum of 1,3-di(1*H*-indol-3-yl)propan-2-one (**1**)	S14
Figure S12	^{1}H-^{13}C-HMBC NMR spectrum of 1,3-di(1*H*-indol-3-yl)propan-2-one (**1**) in $CDCl_3$	S15
Figure S13	Expanded ^{1}H-^{13}C-HMBC NMR spectrum of 1,3-di(1H-indol-3-yl)propan-2-one (**1**)	S16
Figure S14	^{1}H NMR spectrum of 1,3-diphenylpropan-2-one (**25a**) in $CDCl_3$	S17
Figure S15	^{13}C NMR spectrum of 1,3-diphenylpropan-2-one (**25a**) in $CDCl_3$	S18
Figure S16	^{1}H NMR spectrum of 1,3-bis(4-(benzyloxy)phenyl)propan-	S19

	2-one (**25b**) in $CDCl_3$	
Figure S17	^{13}C NMR spectrum of 1,3-bis(4-(benzyloxy)phenyl)propan-2-one (**25b**) in $CDCl_3$	S20
Figure S18	^{1}H NMR spectrum of 1,3-bis(4-hydroxyphenyl)propan-2-one (**25c**) in methanol-d_4	S21
Figure S19	^{13}C NMR spectrum of 1,3-bis(4-hydroxyphenyl)propan-2-one (**25c**) in methanol-d_4	S22
Figure S20	^{1}H NMR spectrum of 1,3-bis(1-benzyl-1*H*-indol-3-yl)propan-2-one (**25d**) in $CDCl_3$	S23
Figure S21	^{13}C NMR spectrum of 1,3-bis(1-benzyl-1*H*-indol-3-yl)propan-2-one (**25d**) in $CDCl_3$	S24
Figure S22	High-resolution mass spectrum of 2-(1-(*tert*-butoxycarbonyl)-1*H*-indol-3-yl)acetic acid (**22**)	S25
Figure S23	High-resolution mass spectrum of di-*tert*-butyl 3,3'-(2-oxo-propane-1,3-diyl)bis(1*H*-indole-1-carboxylate) (**23**)	S26
Figure S24	High resolution mass spectrum of 1,3-di(1*H*-indol-3-yl)propan-2-one (**1**)	S27
Figure S25	High resolution mass spectrum of 1,3-bis(4-(benzyloxy)-phenyl)propan-2-one (**25b**)	S28
Figure S26	High resolution mass spectrum of 1,3-bis(4-hydroxyphenyl)-propan-2-one (**25c**)	S29
Figure S27	High-resolution mass spectrum of 1,3-bis(1-benzyl-1*H*-indol-2-yl)propan-2-one (**25d**)	S30

Figure S1. ^{1}H NMR spectrum of *tert*-butyl 3-(2-methoxy-2-oxoethyl)-1*H*-indole-1-carboxylate (**21**) in $CDCl_3$

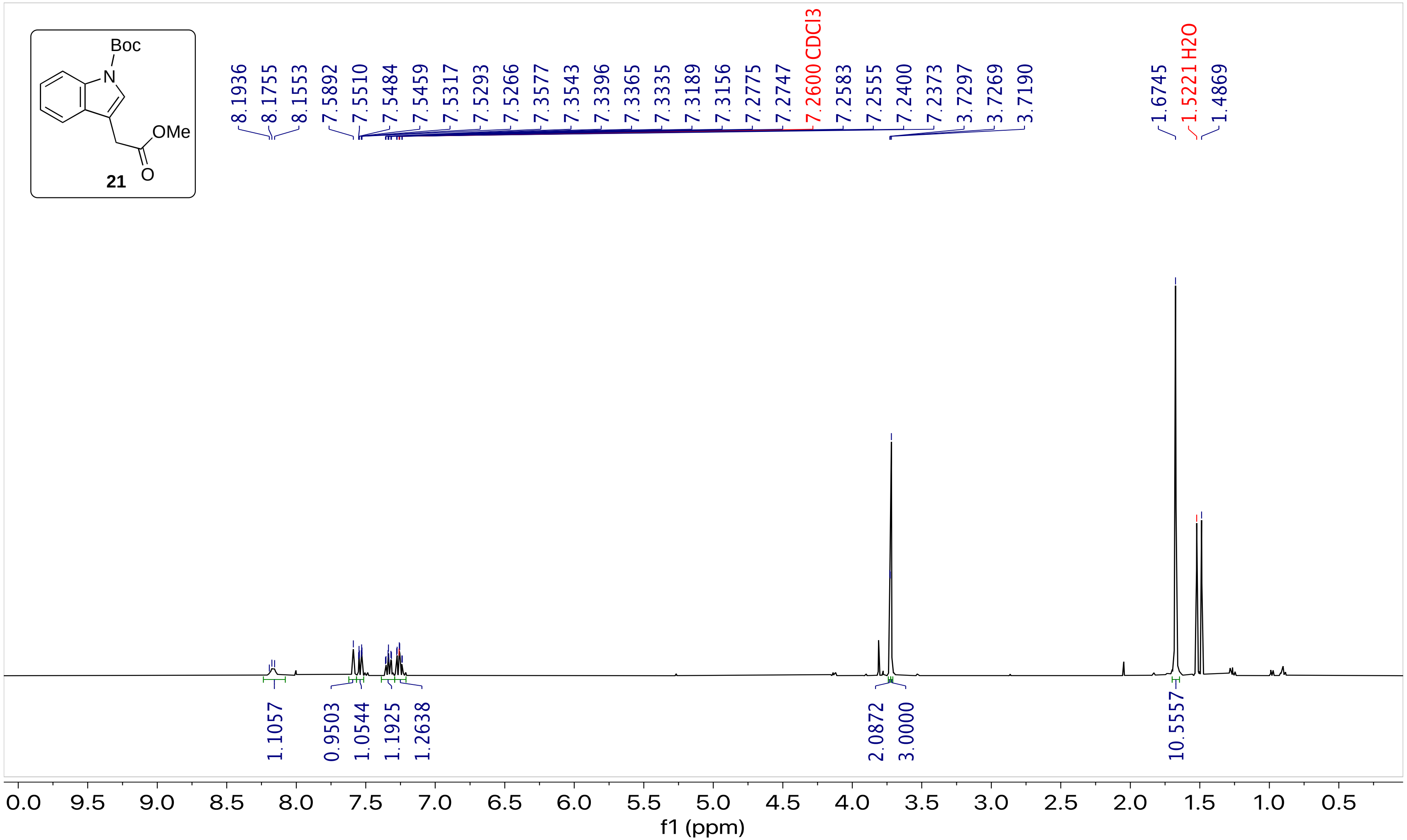

Figure S2. ^{13}C NMR spectrum of *tert*-butyl 3-(2-methoxy-2-oxoethyl)-1*H*-indole-1-carboxylate (**21**) in $CDCl_3$

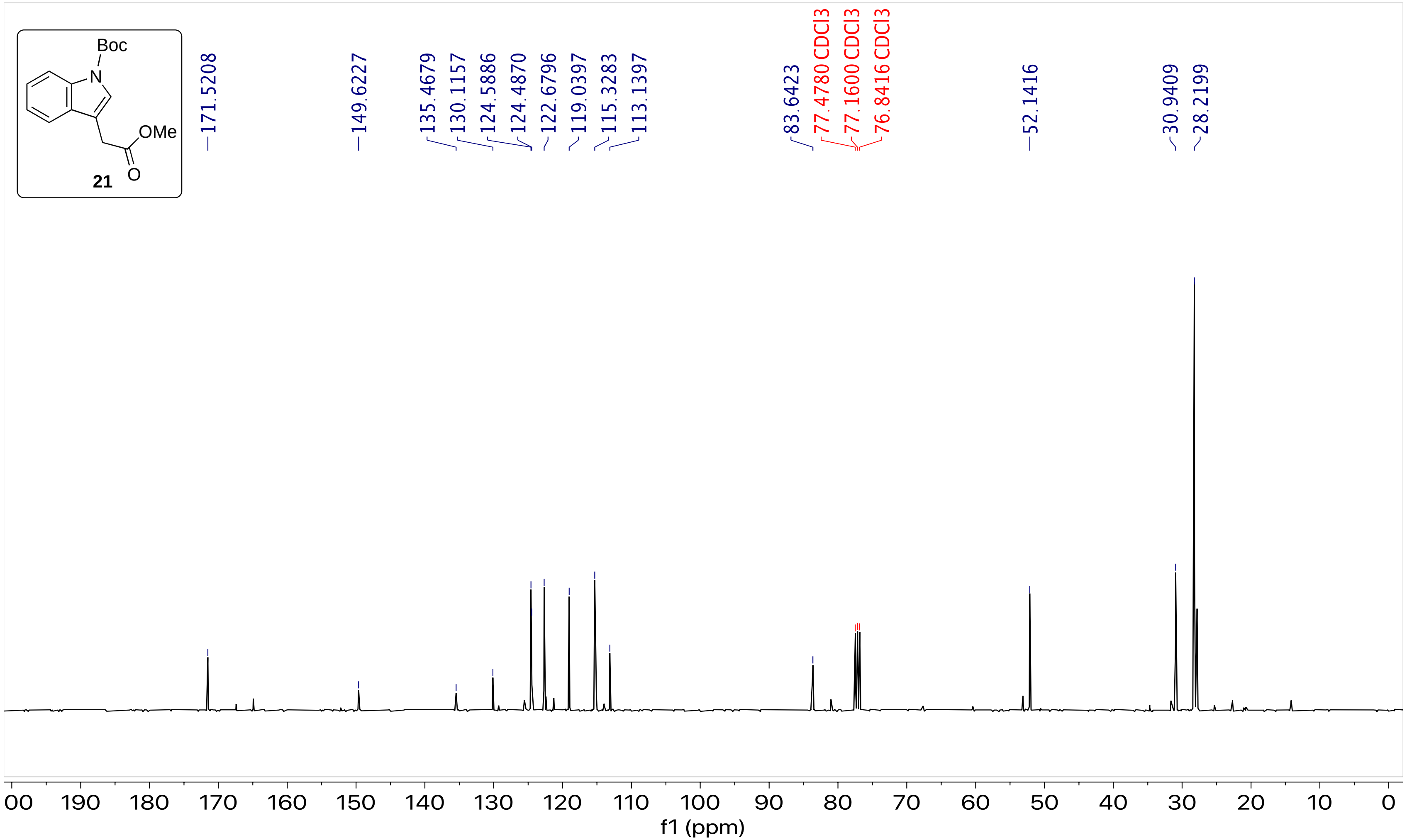

Figure S3. ^{1}H NMR spectrum of 2-(1-(*tert*-butoxycarbonyl)-1*H*-indol-3-yl)acetic acid (**22**) in $CDCl_3$

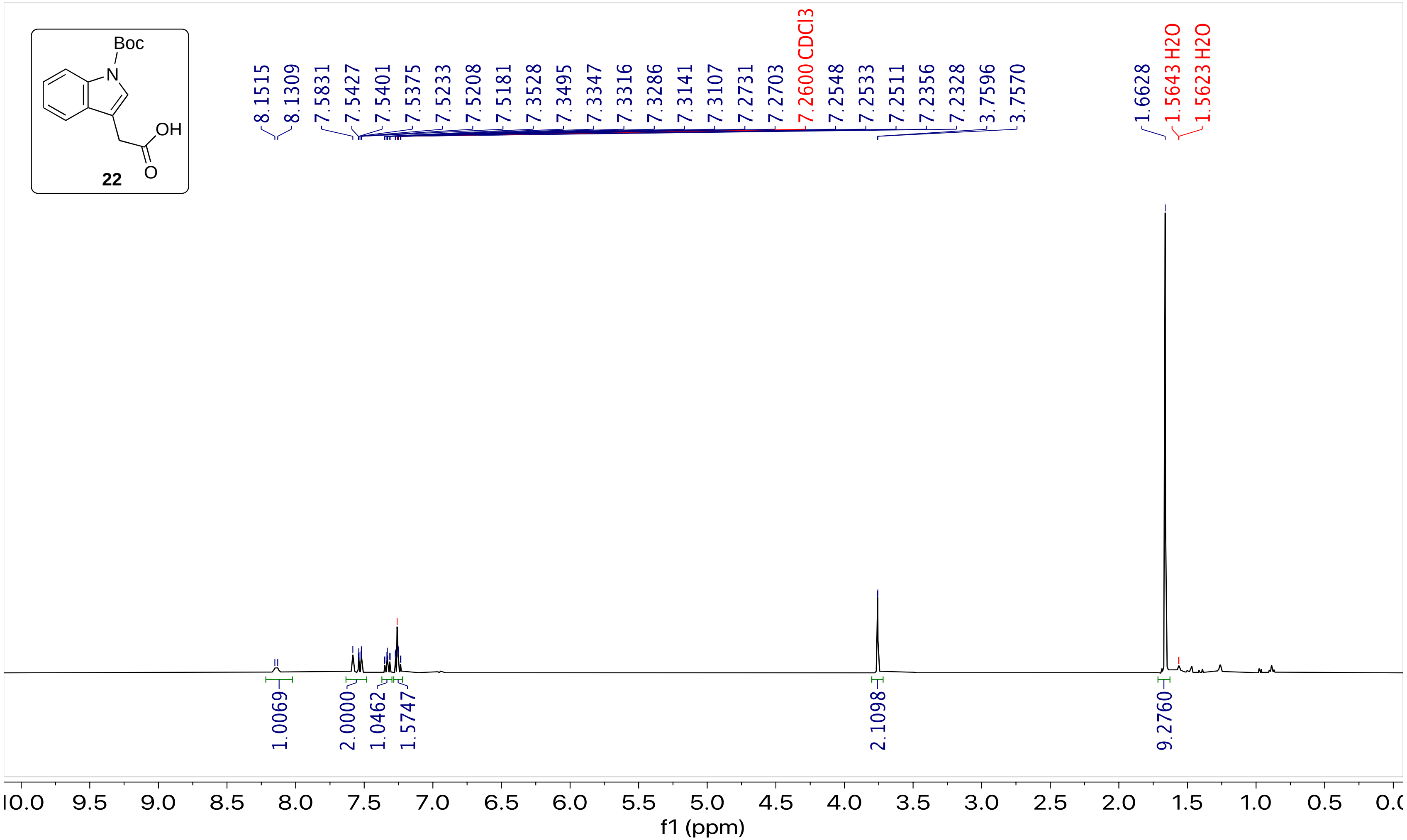

Figure S4. ^{13}C NMR spectrum of 2-(1-(*tert*-butoxycarbonyl)-1*H*-indol-3-yl)acetic acid (**22**) in $CDCl_3$

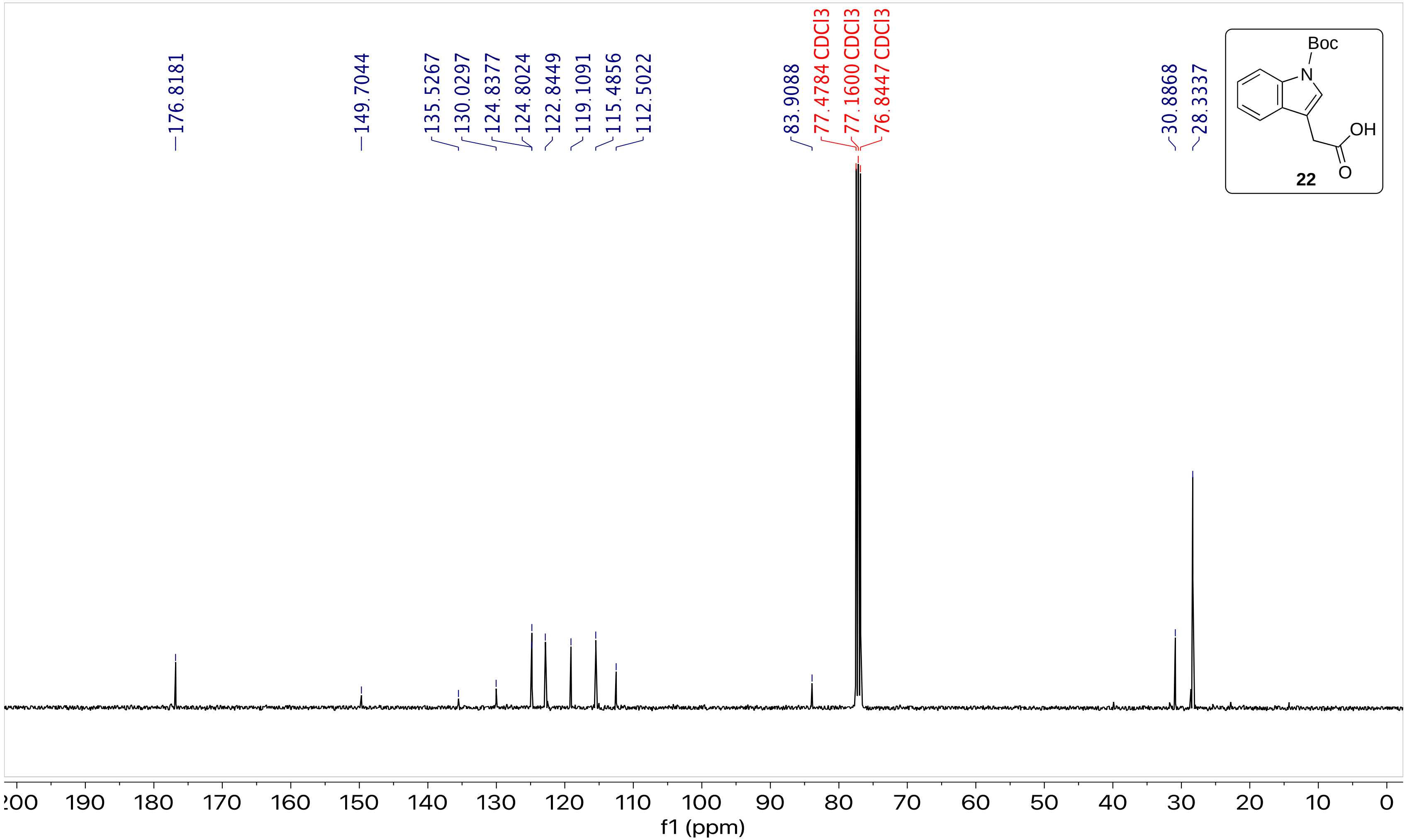

Figure S5. ^{1}H NMR spectrum of di-*tert*-butyl 3,3'-(2-oxopropane-1,3-diyl)bis(1*H*-indole-1-carboxylate) (**23**) in $CDCl_3$

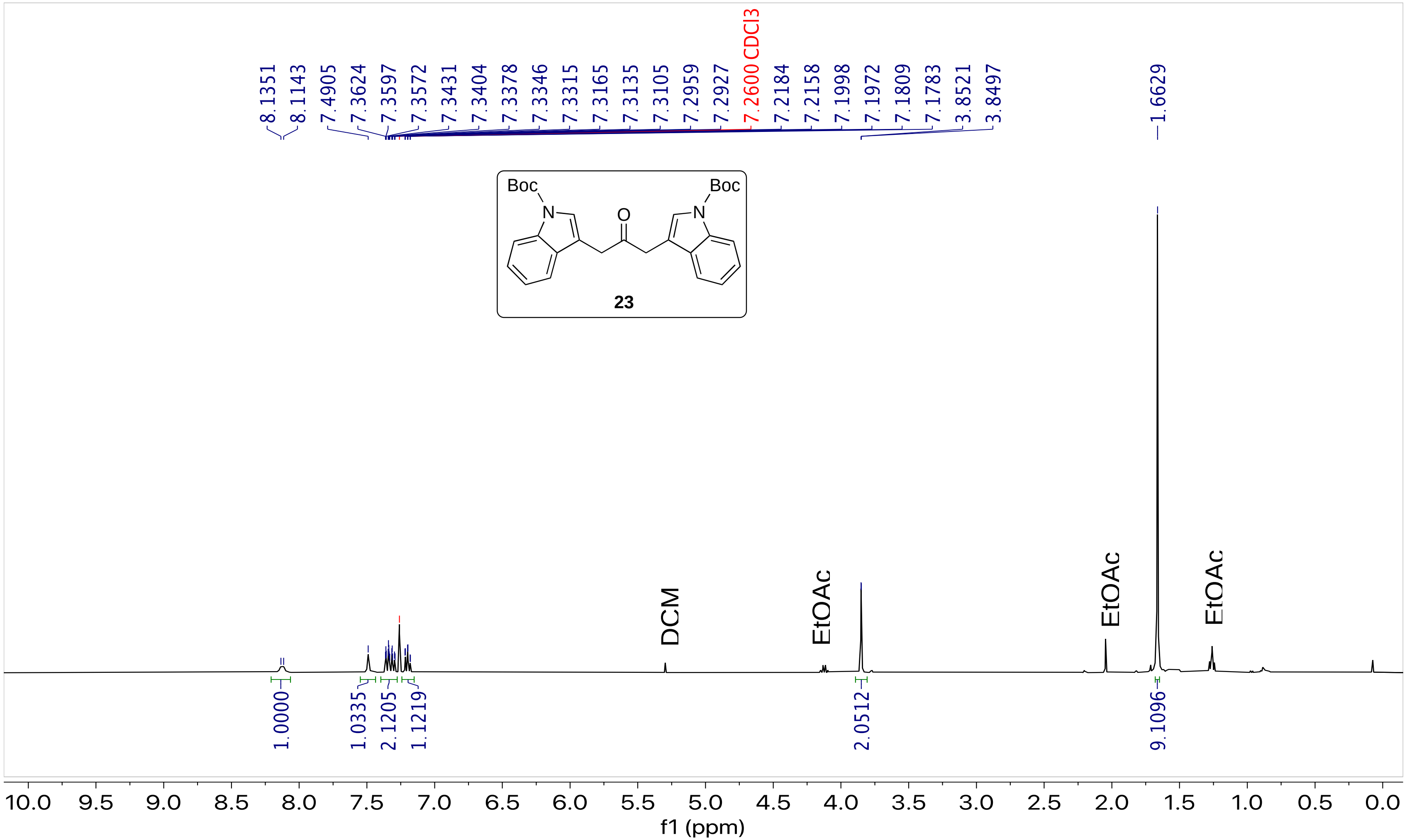

Figure S6. ^{13}C NMR spectrum of di-*tert*-butyl 3,3'-(2-oxopropane-1,3-diyl)bis(1*H*-indole-1-carboxylate) (**23**) in CDCl₃

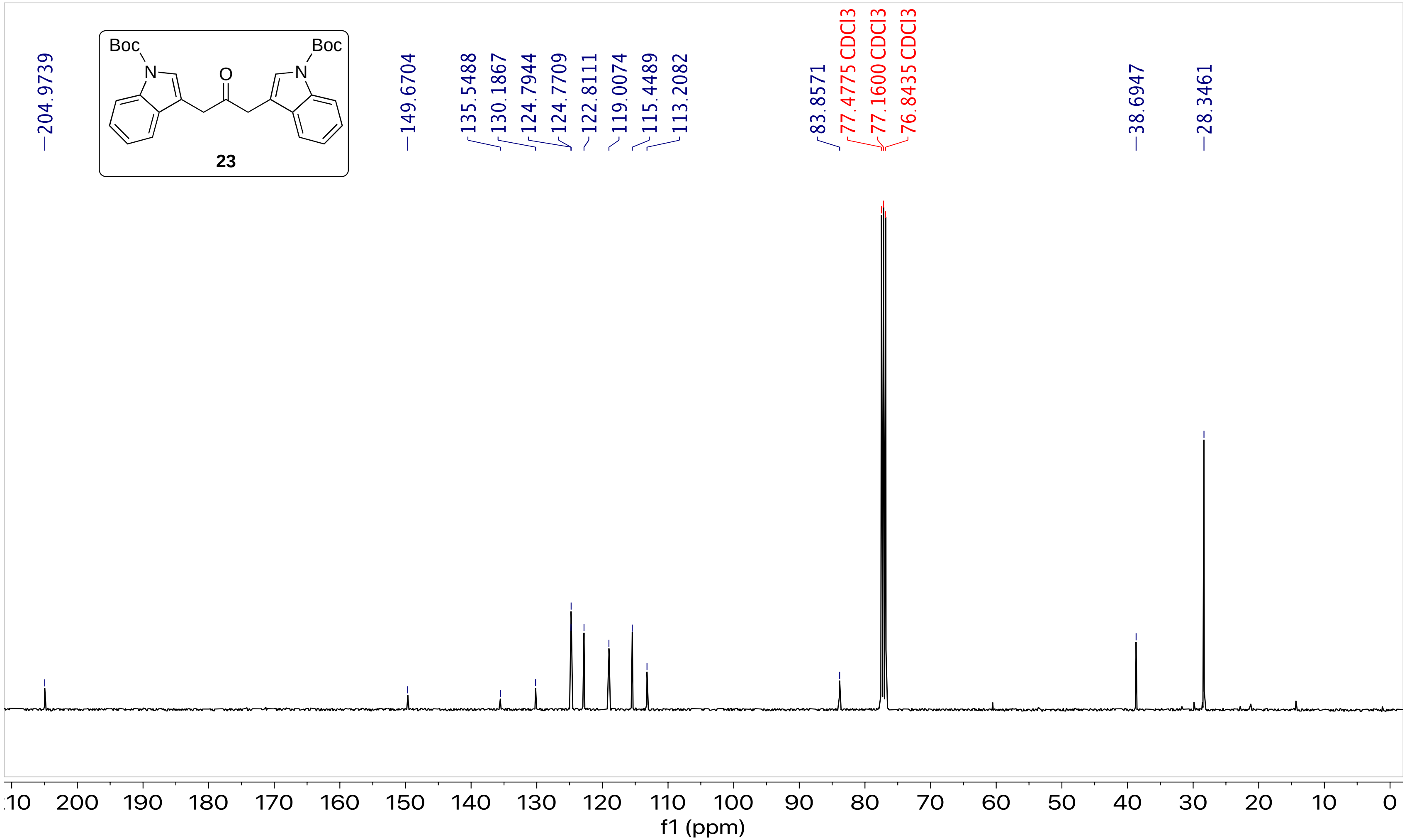

Figure S7. ^{1}H NMR spectrum of 1,3-di(1*H*-indol-3-yl)propan-2-one (**1**) in $CDCl_3$

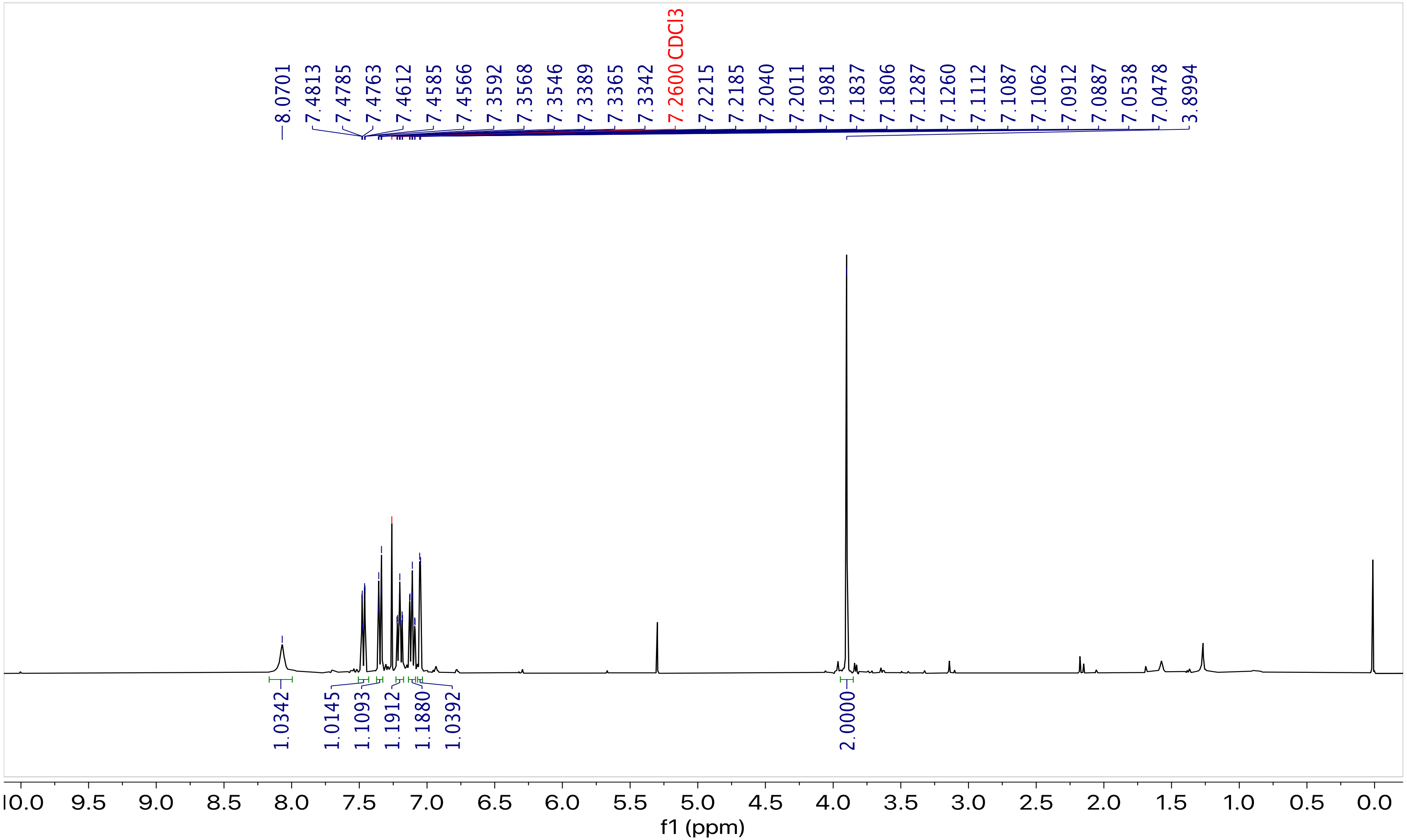

Figure S8. ^{13}C NMR spectrum of 1,3-di(1*H*-indol-3-yl)propan-2-one (**1**) in $CDCl_3$

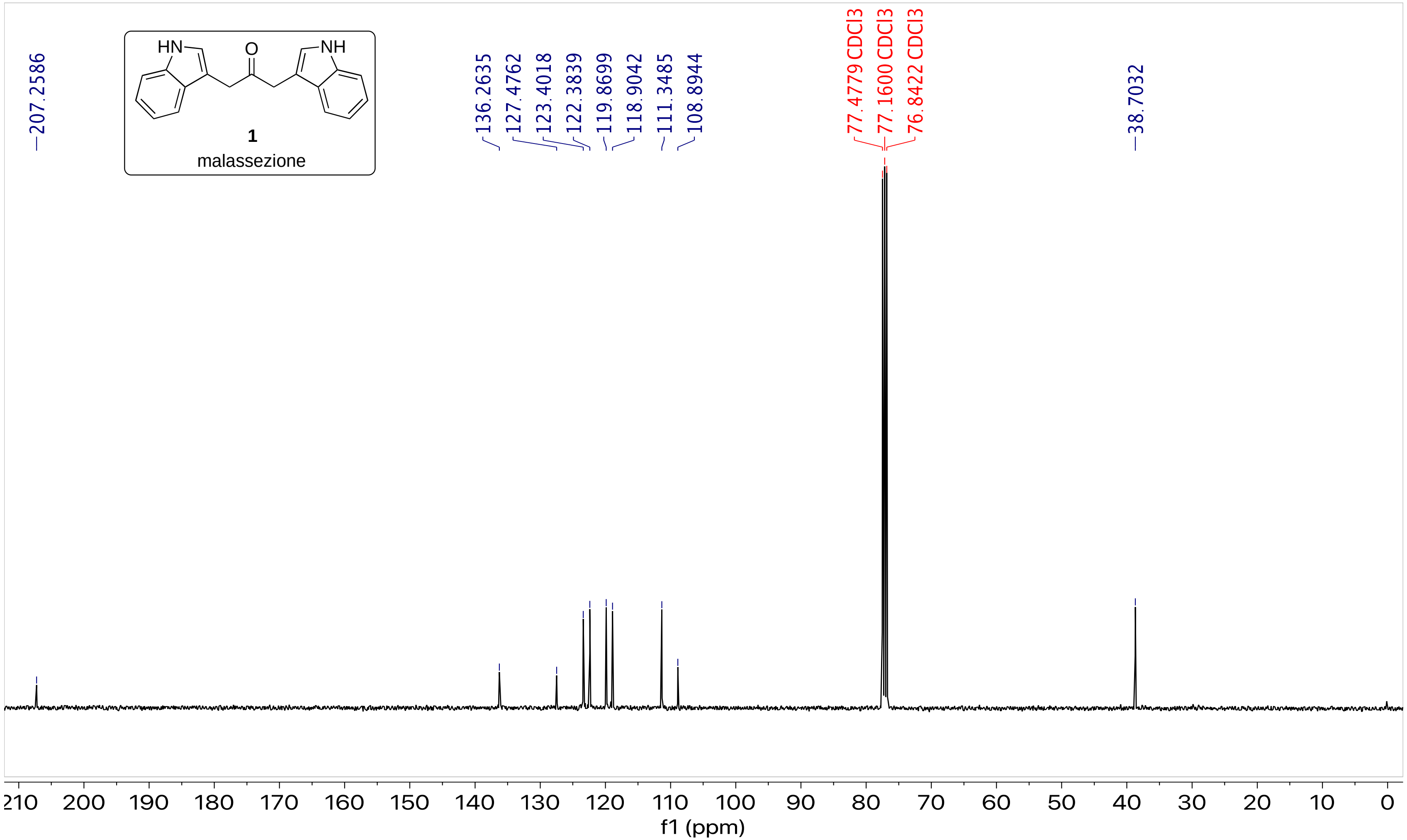

Figure S9. ^{1}H-^{1}H-COSY NMR spectrum of 1,3-di(1*H*-indol-3-yl)propan-2-one (**1**) in $CDCl_3$

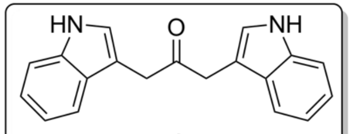

1
malassezione

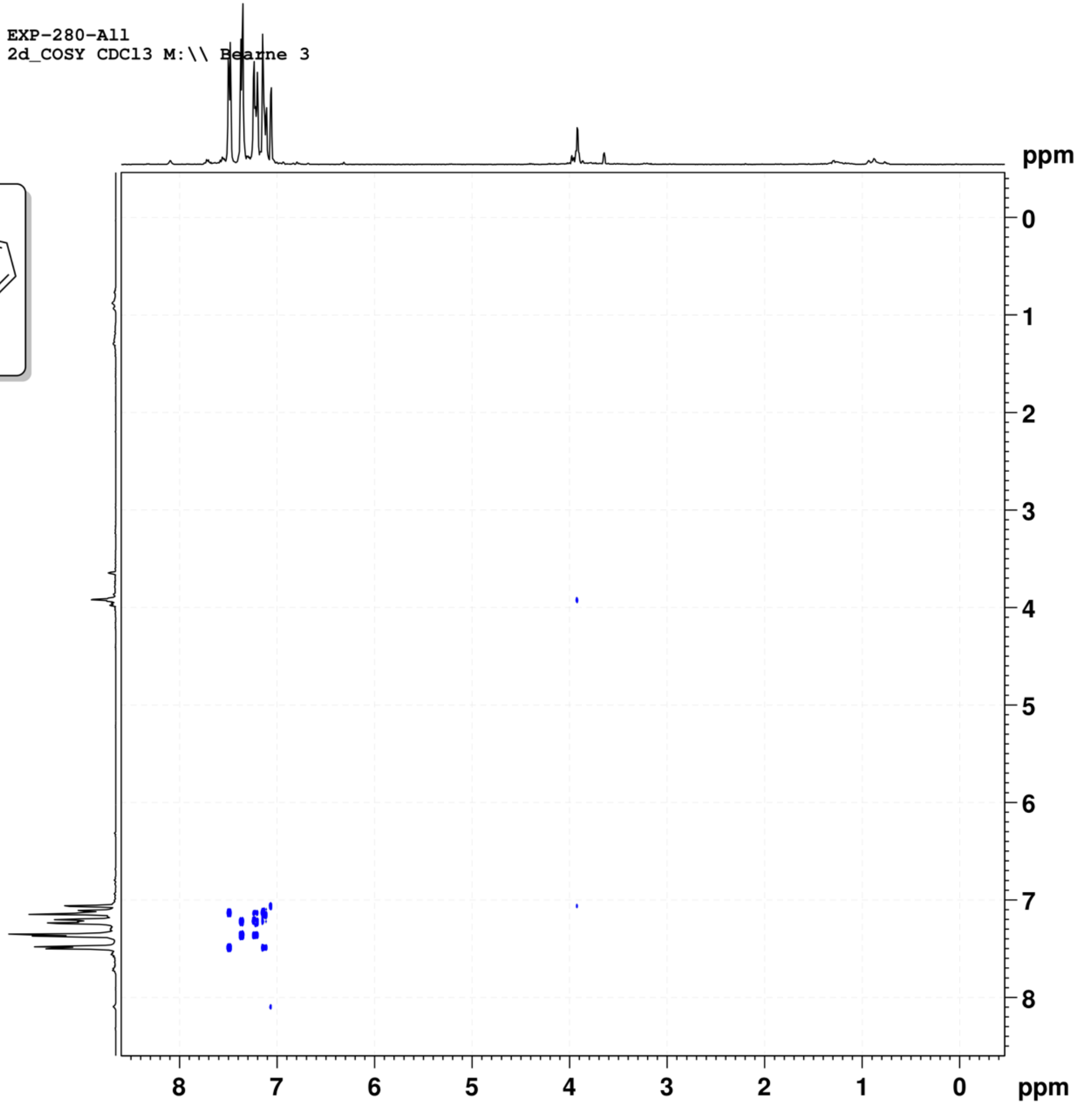


```
Current Data Parameters
NAME     Jan06-2025-Bearne-EXP-279 Jan-6
EXPNO                   2
PROCNO                  1

F2 - Acquisition Parameters
Date_            20250106
Time                20.49 h
INSTRUM  Avance Neo Nanobay
PROBHD   Z163739_0636 (
PULPROG    cosygpmfppqf
TD                   4096
SOLVENT             CDCl3
NS                     32
DS                     16
SWH              5555.556 Hz
FIDRES           2.712674 Hz
AQ              0.3686400 sec
RG                    101
DW                 90.000 usec
DE                  11.25 usec
TE                  298.0 K
D0                      0 sec
D1             0.75000000 sec
D11            0.03000000 sec
D12            0.00002000 sec
D13            0.00000400 sec
D16            0.00020000 sec
IN0            0.00018000 sec
TDav                    1
SFO1          400.3024018 MHz
NUC1                   1H
P1                   7.70 usec
P17               2500.00 usec
PLW1          25.00000000 W
PLW10          1.64690006 W
GPNAM[1]       SMSQ10.100
GPZ1                16.00 %
GPNAM[2]       SMSQ10.100
GPZ2                12.00 %
GPNAM[3]       SMSQ10.100
GPZ3                40.00 %
P16               1000.00 usec

======== F1 INDIRECT DIMENSION ========
td1      256
sw_F1    13.878398

F1 - Acquisition parameters
TD                    256
SFO1             400.3024 MHz
FIDRES          43.402782 Hz
SW                 13.878 ppm
FnMODE                 QF

F2 - Processing parameters
SI                   2048
SF            400.3000000 MHz
WDW                 QSINE
SSB                     4
LB                      0 Hz
GB                      0
PC                   1.00

F1 - Processing parameters
SI                   2048
MC2                    QF
SF            400.3000000 MHz
WDW                 QSINE
SSB                     4
LB                      0 Hz
GB                      0
```

Figure S10. Expanded ^{1}H-^{1}H-COSY NMR spectrum of 1,3-di(1*H*-indol-3-yl)propan-2-one (**1**)

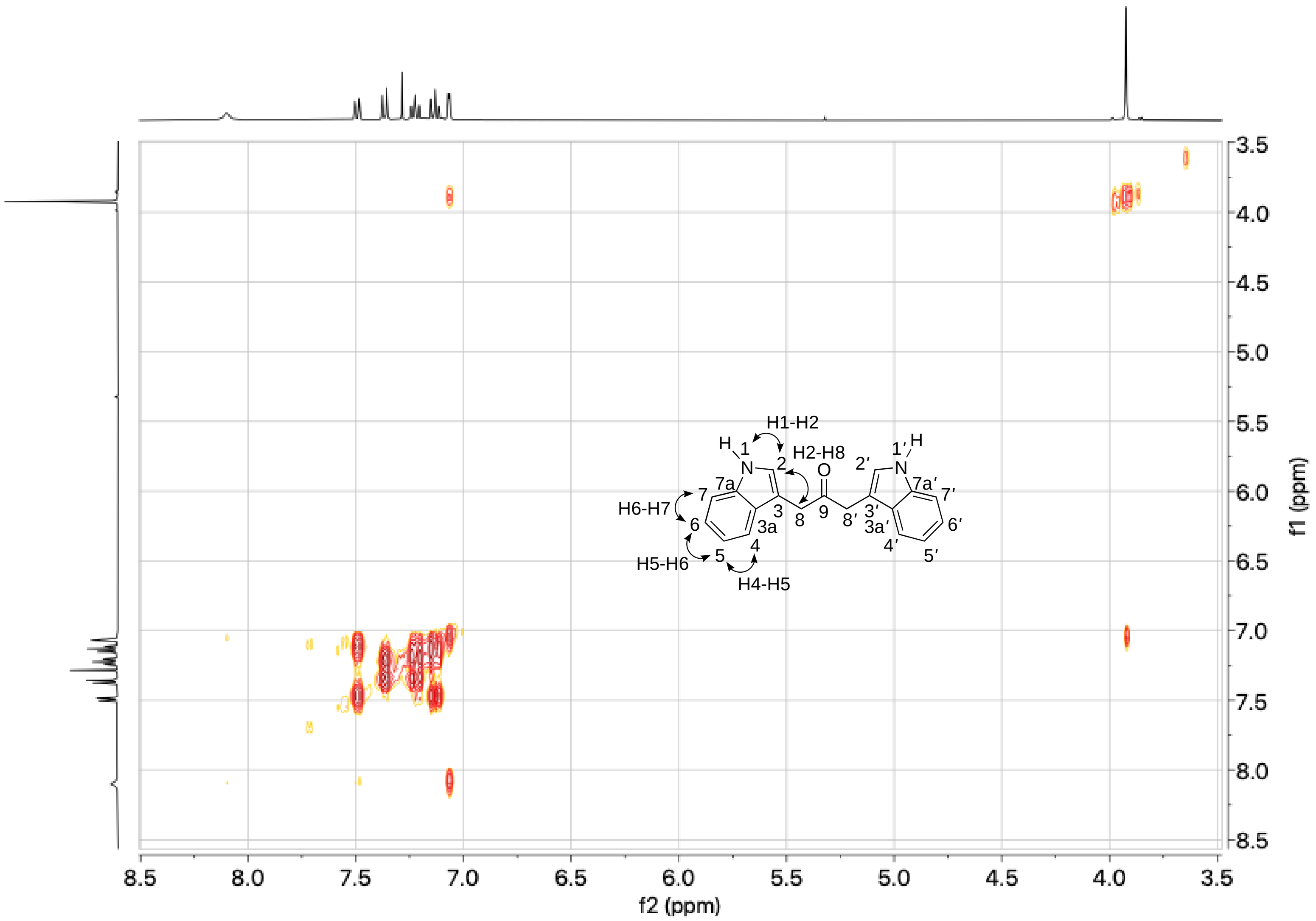

Figure S11. Expanded aromatic region of the ^{1}H-^{1}H-COSY NMR spectrum of 1,3-di(1*H*-indol-3-yl)propan-2-one (**1**)

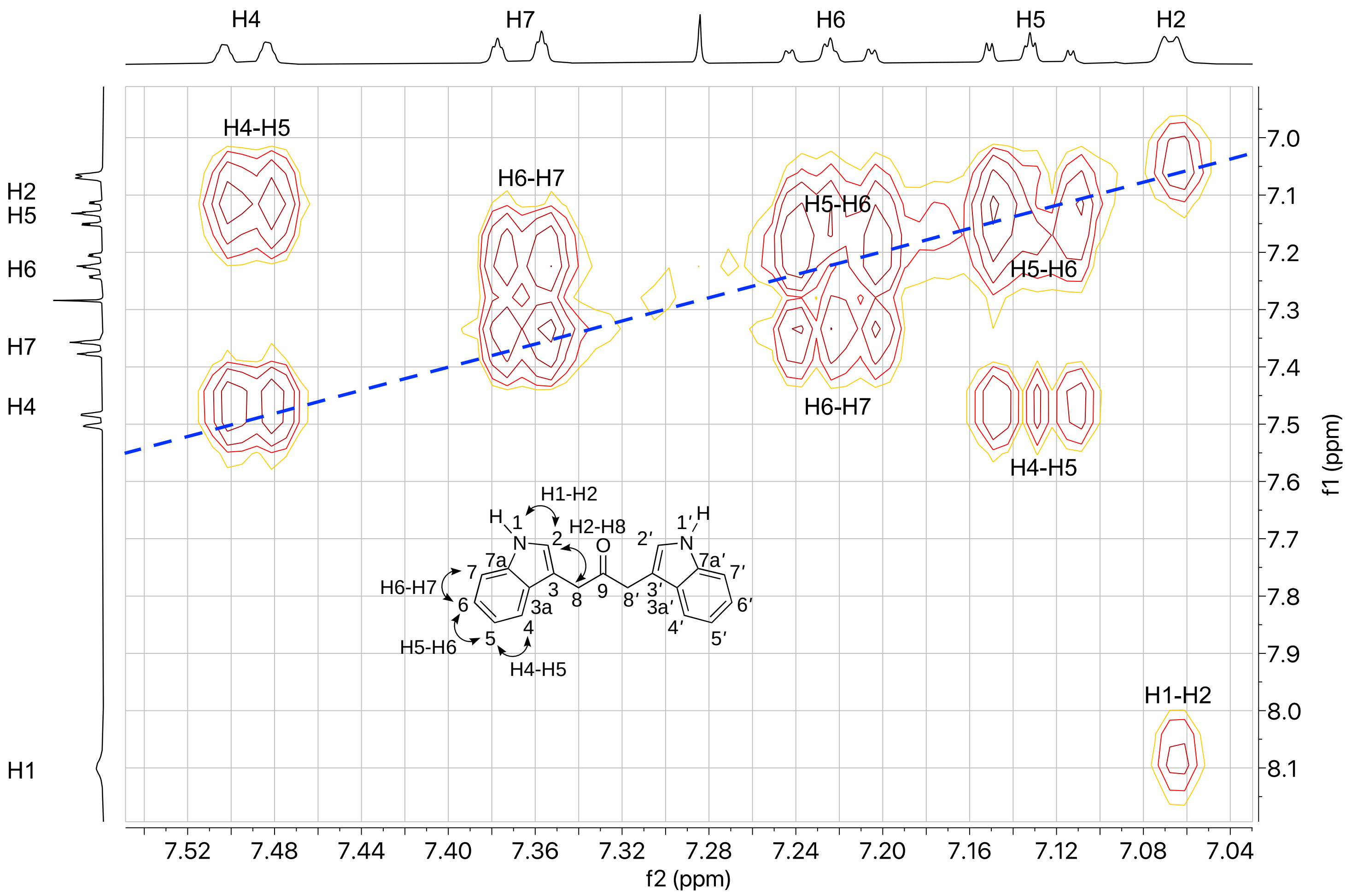

Figure S12. ^{1}H-^{13}C-HMBC NMR spectrum of 1,3-di(1*H*-indol-3-yl)propan-2-one (**1**) in $CDCl_3$

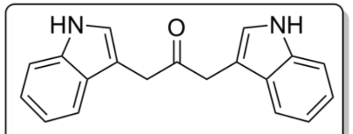

1
malassezione

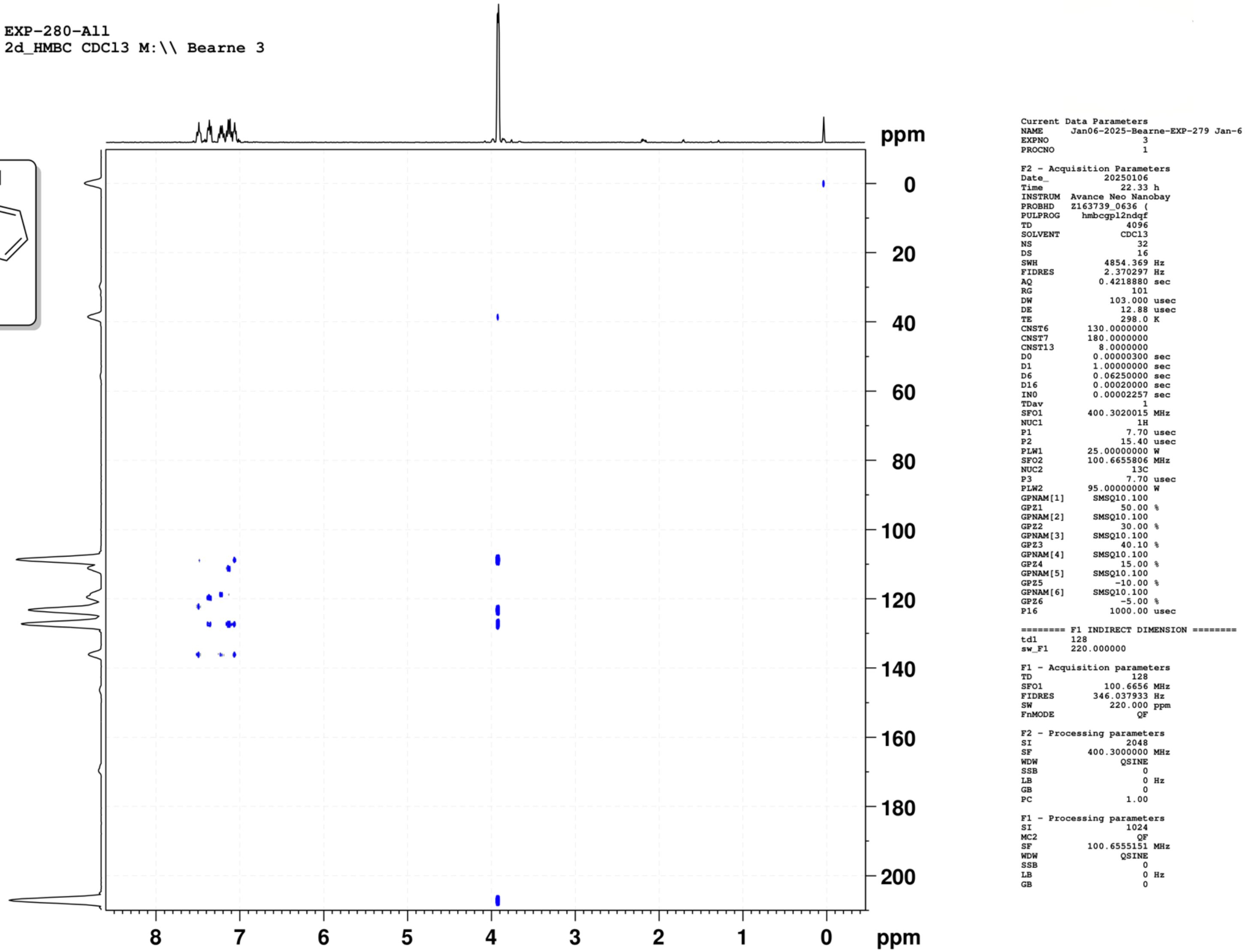

Figure S13. Expanded ^{1}H-^{13}C-HMBC NMR spectrum of 1,3-di(1*H*-indol-3-yl)propan-2-one (**1**)

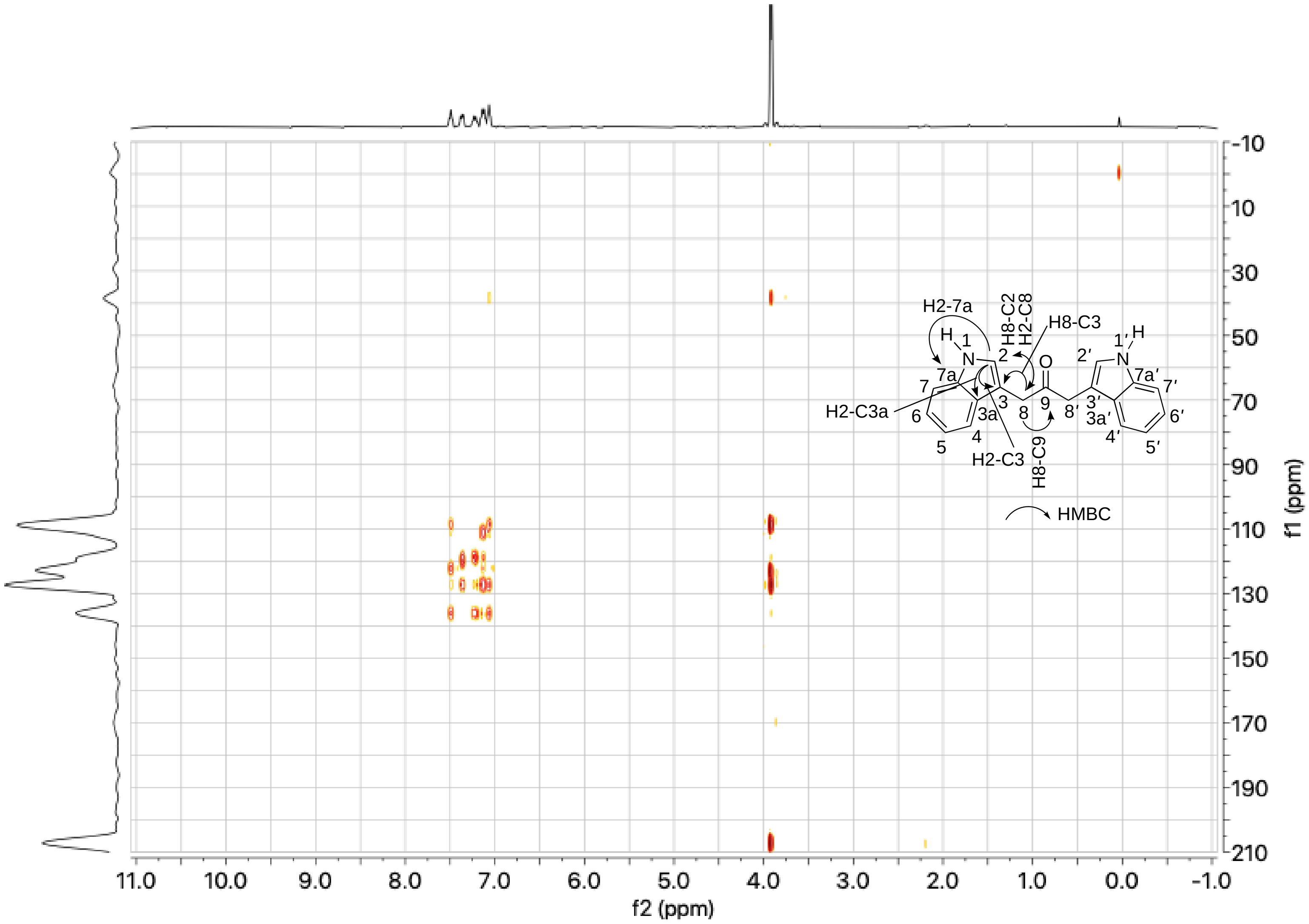

Figure S14. ^{1}H NMR spectrum of 1,3-diphenylpropan-2-one (**25a**) in $CDCl_3$

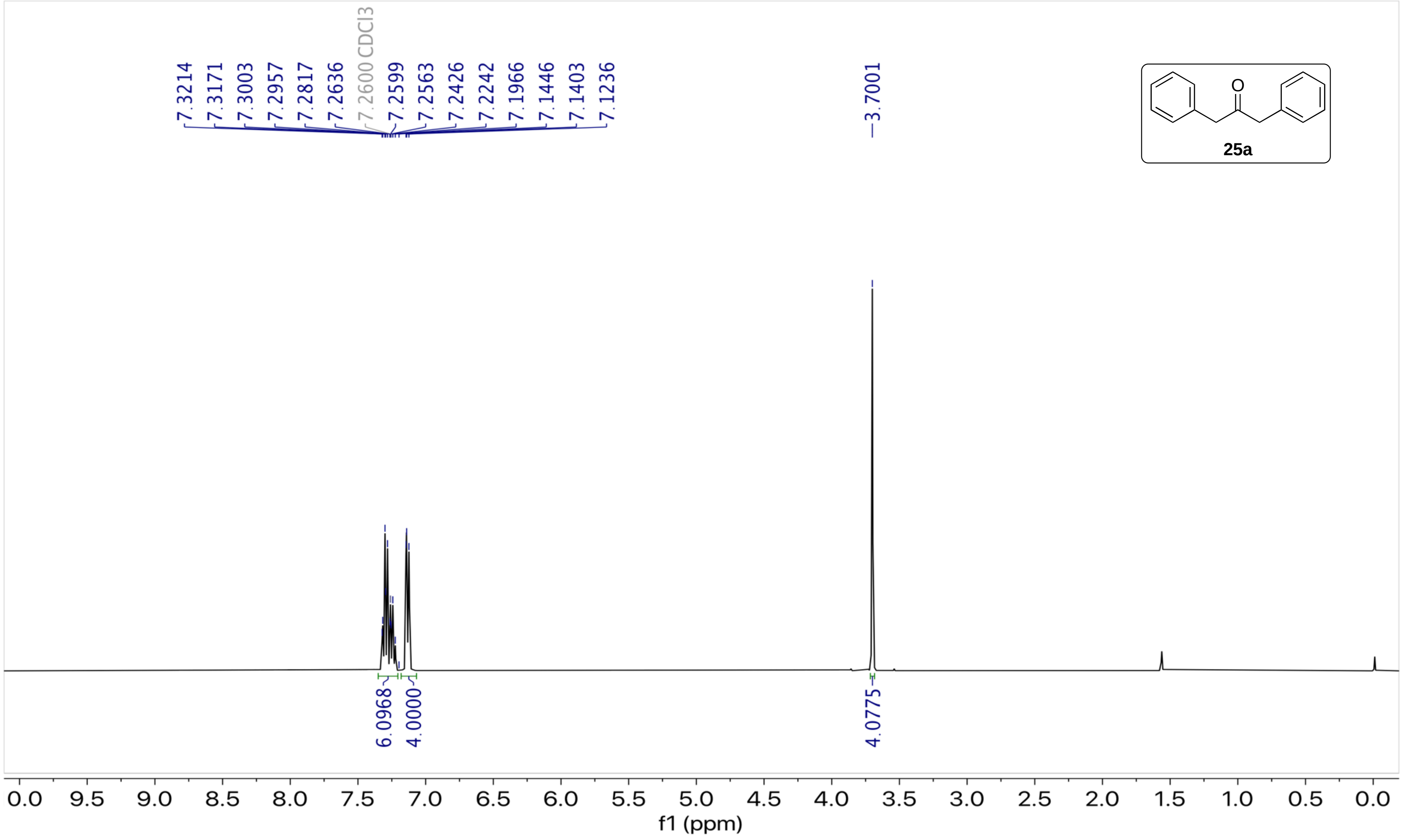

Figure S15. ^{13}C NMR spectrum of 1,3-diphenylpropan-2-one (**25a**) in $CDCl_3$

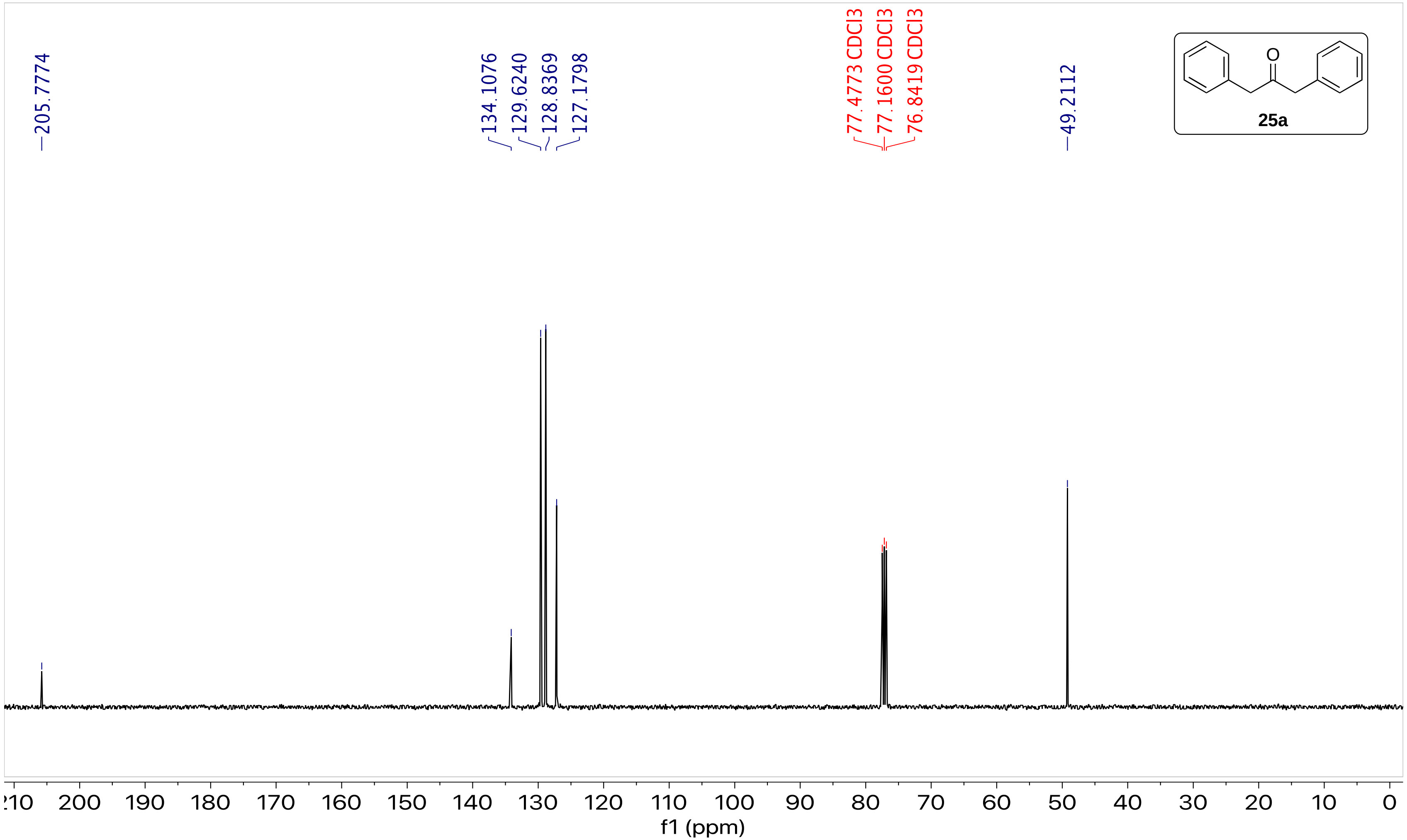

Figure S16. ^{1}H NMR spectrum of 1,3-bis(4-(benzyloxy)phenyl)propan-2-one (**25b**) in $CDCl_3$

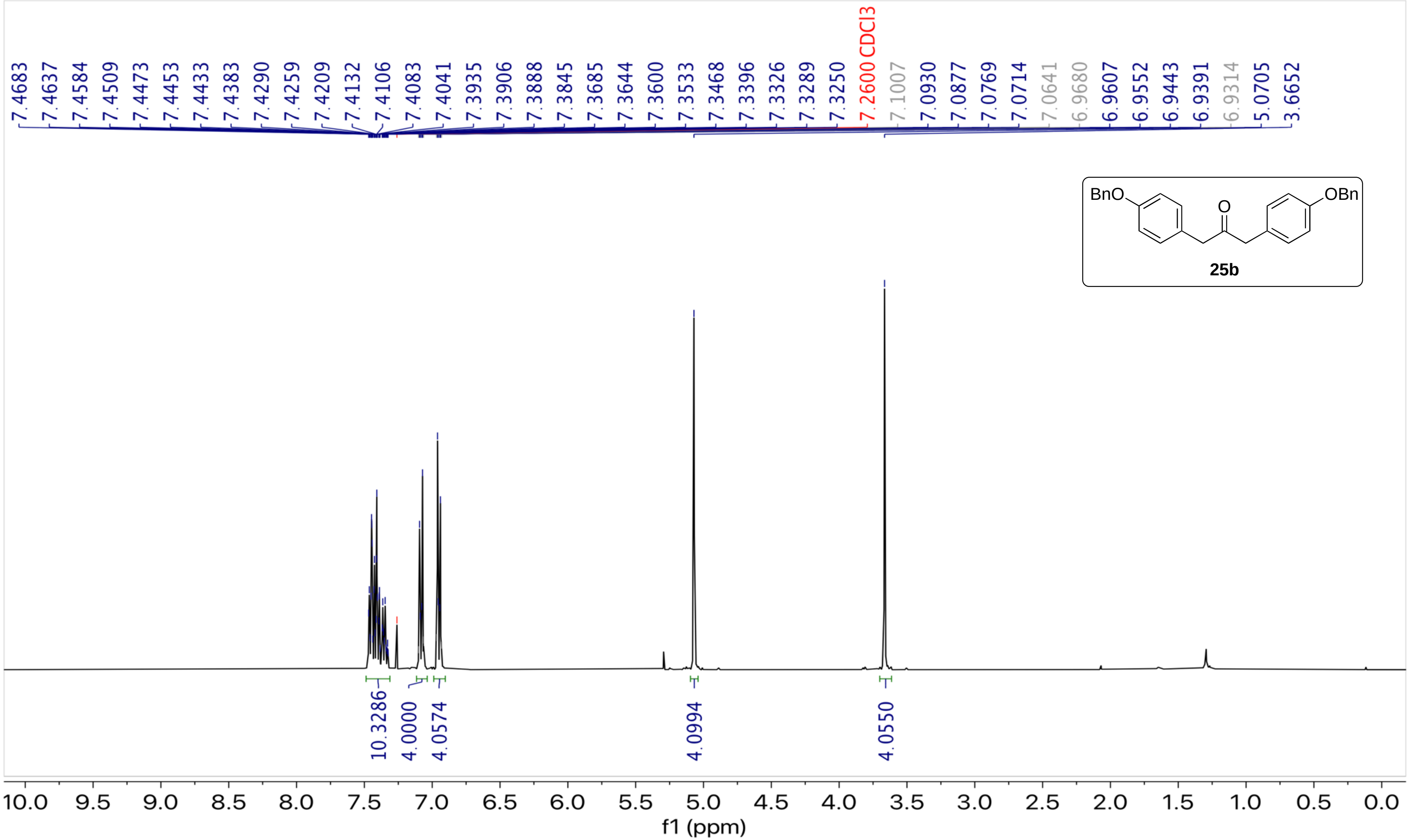

Figure S17. ^{13}C NMR spectrum of 1,3-bis(4-(benzyloxy)phenyl)propan-2-one (**25b**) in $CDCl_3$

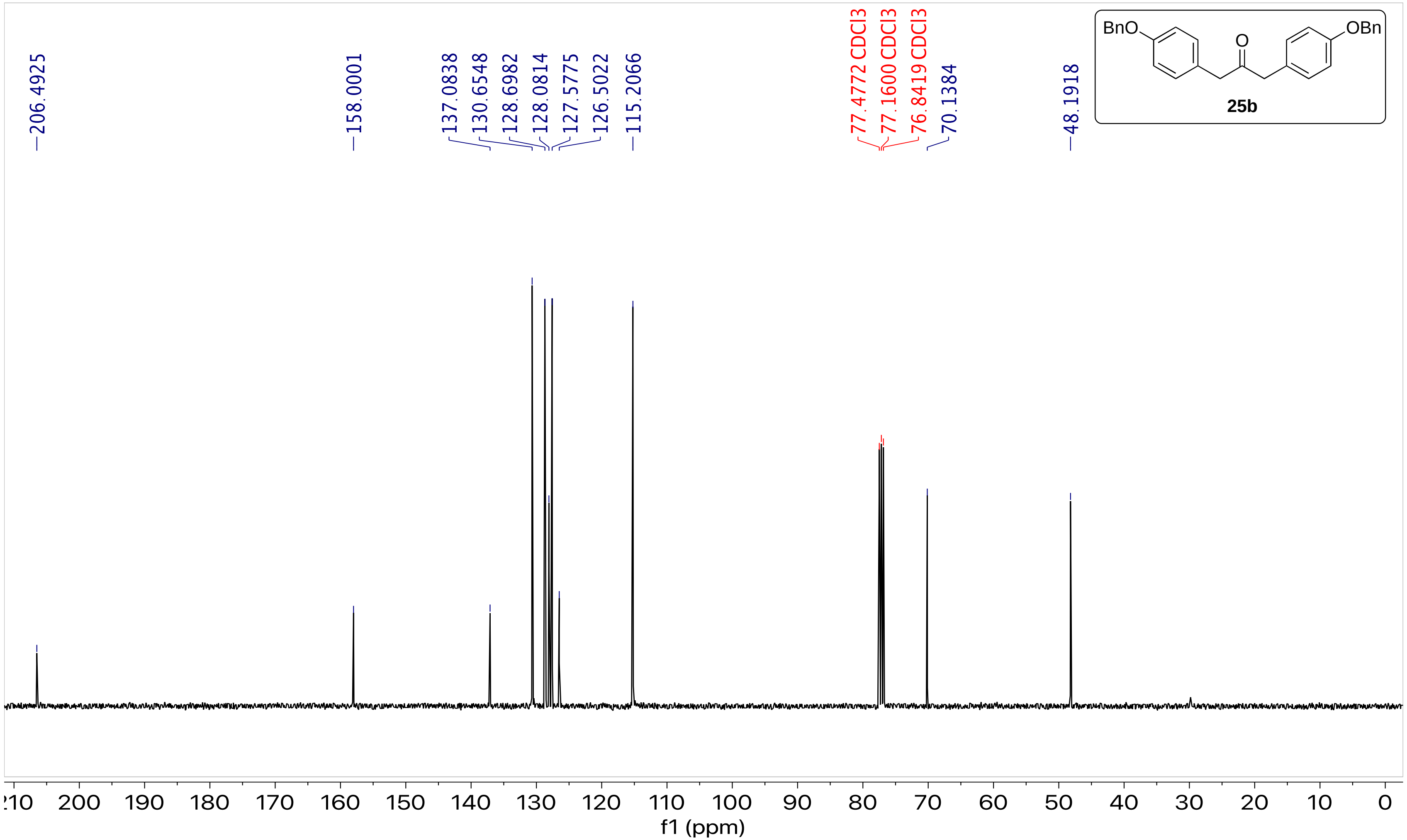

Figure S18. ^{1}H NMR spectrum of 1,3-bis(4-hydroxyphenyl)propan-2-one (**25c**) in methanol-d_4

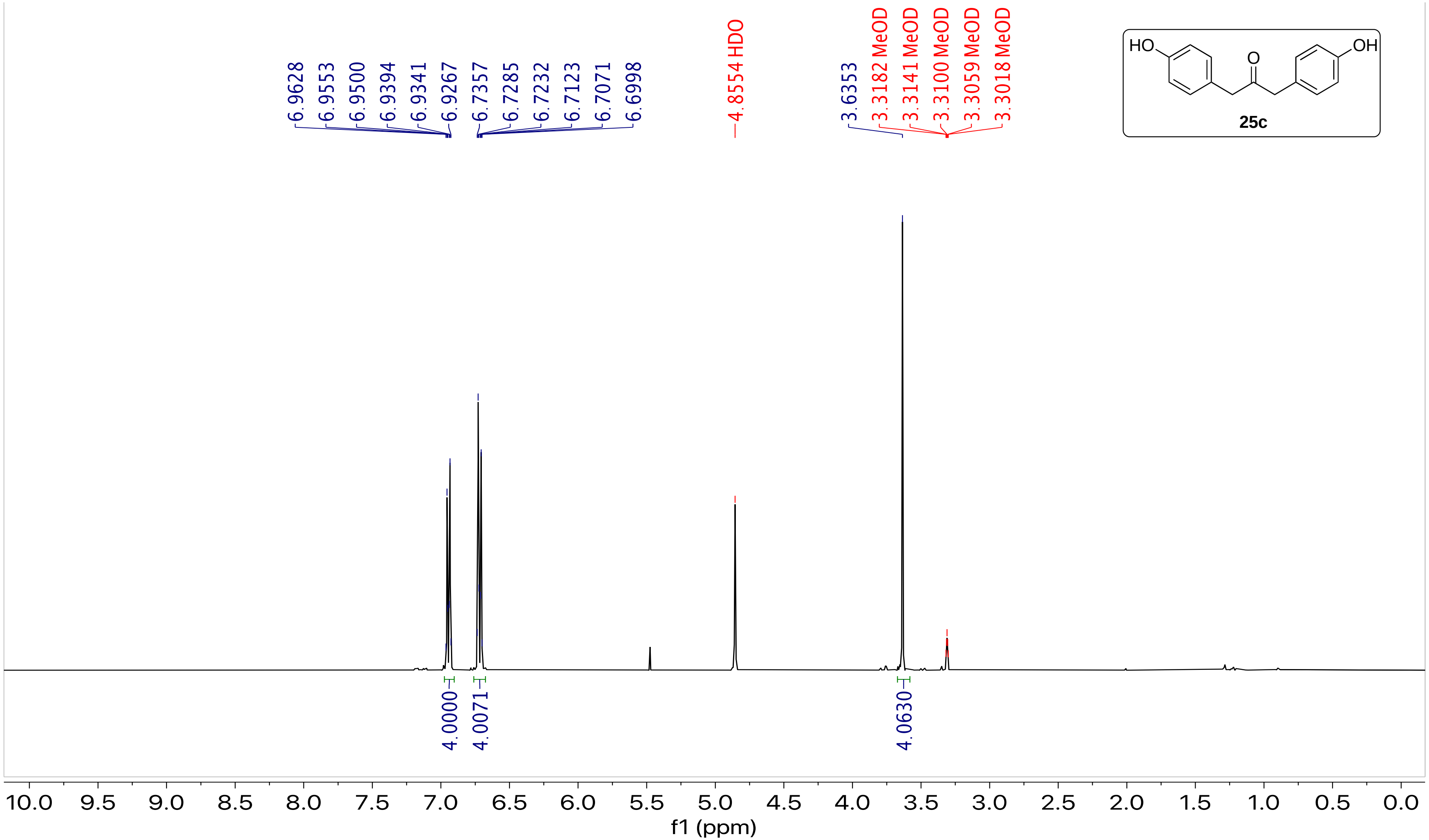

Figure S19. ^{13}C NMR spectrum of 1,3-bis(4-hydroxyphenyl)propan-2-one (**25c**) in methanol-d_4

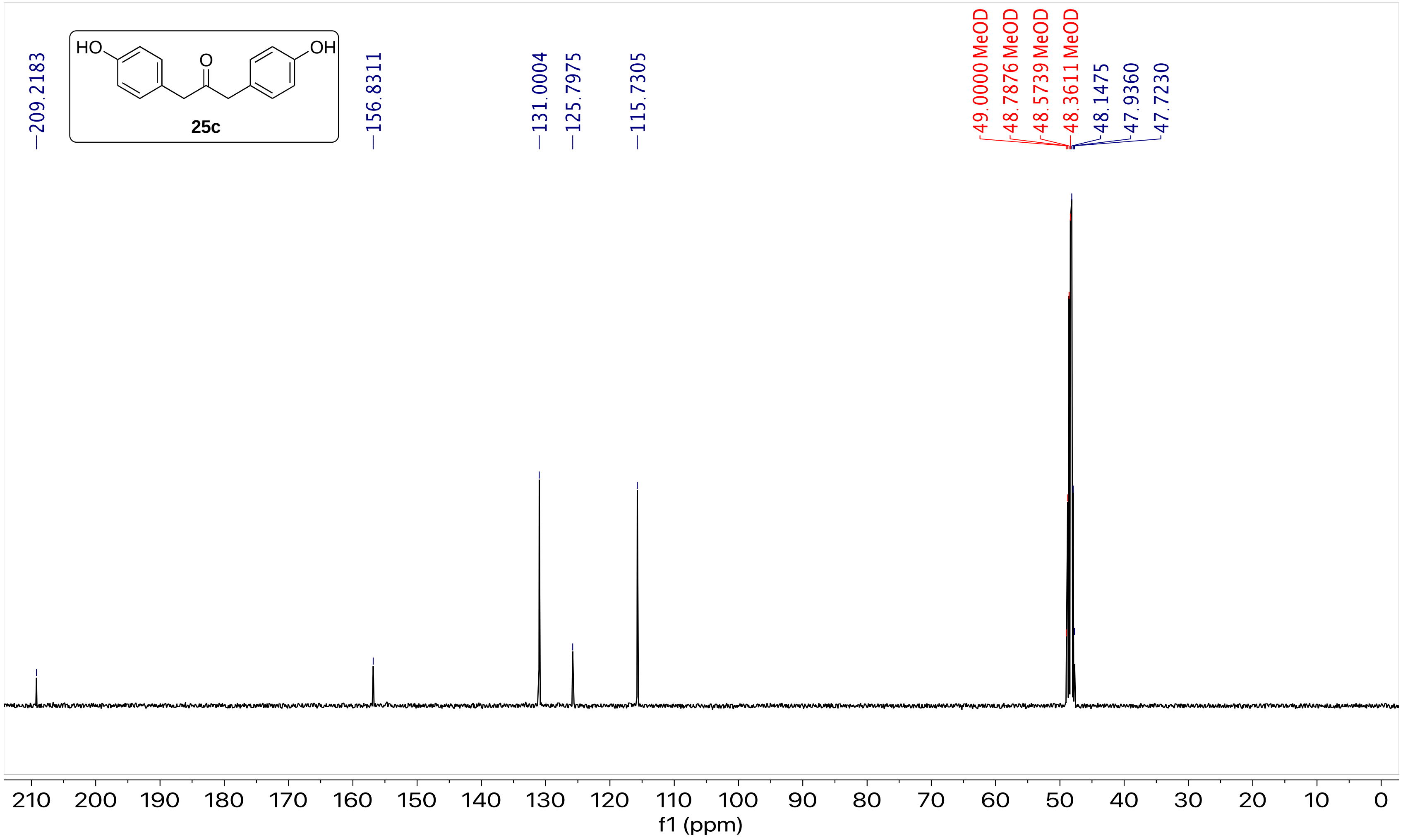

Figure S20. ^{1}H NMR spectrum of 1,3-bis(1-benzyl-1*H*-indol-3-yl)propan-2-one (**25d**) in $CDCl_3$

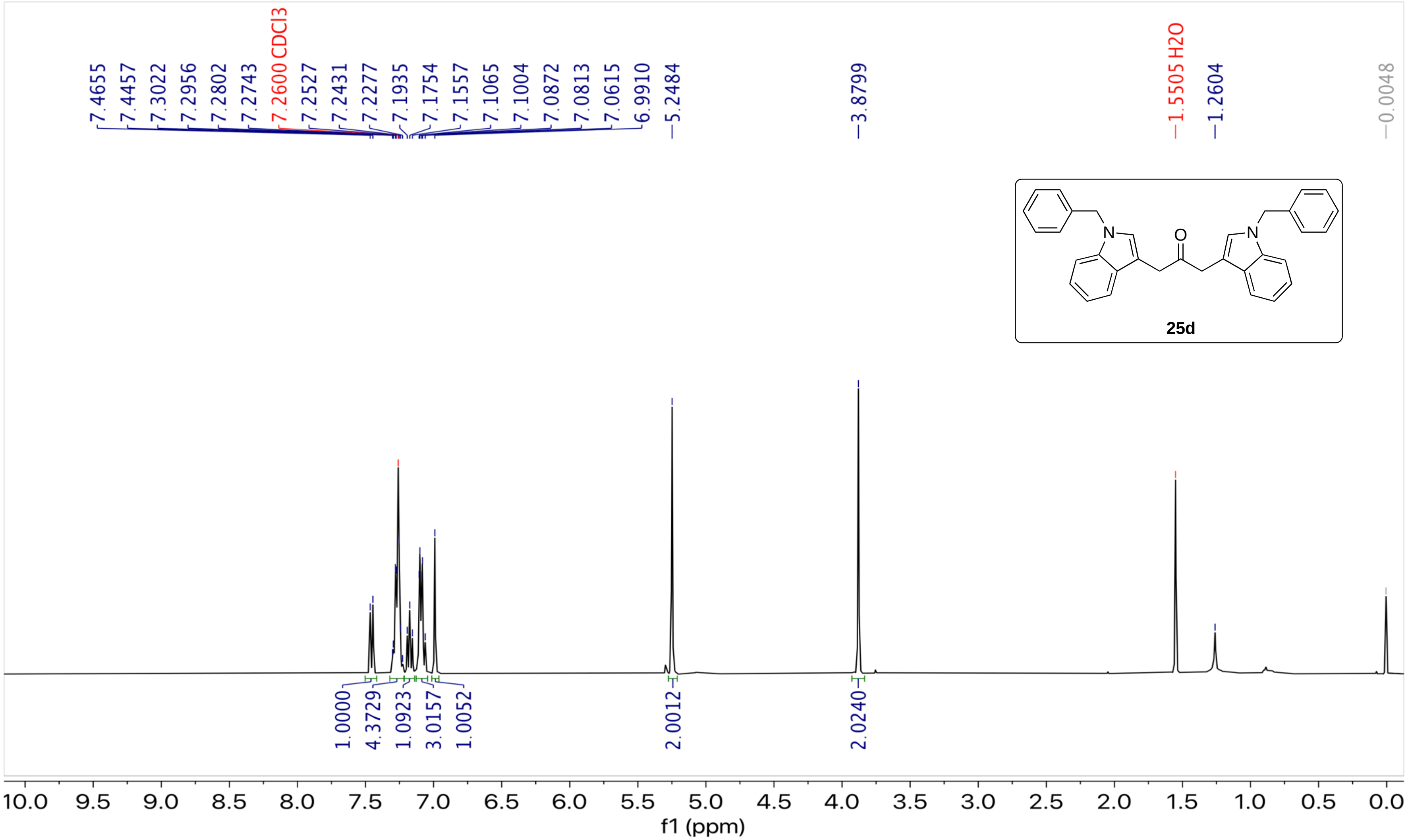

Figure S21. ^{13}C NMR spectrum of 1,3-bis(1-benzyl-1*H*-indol-3-yl)propan-2-one (**25d**) in $CDCl_3$

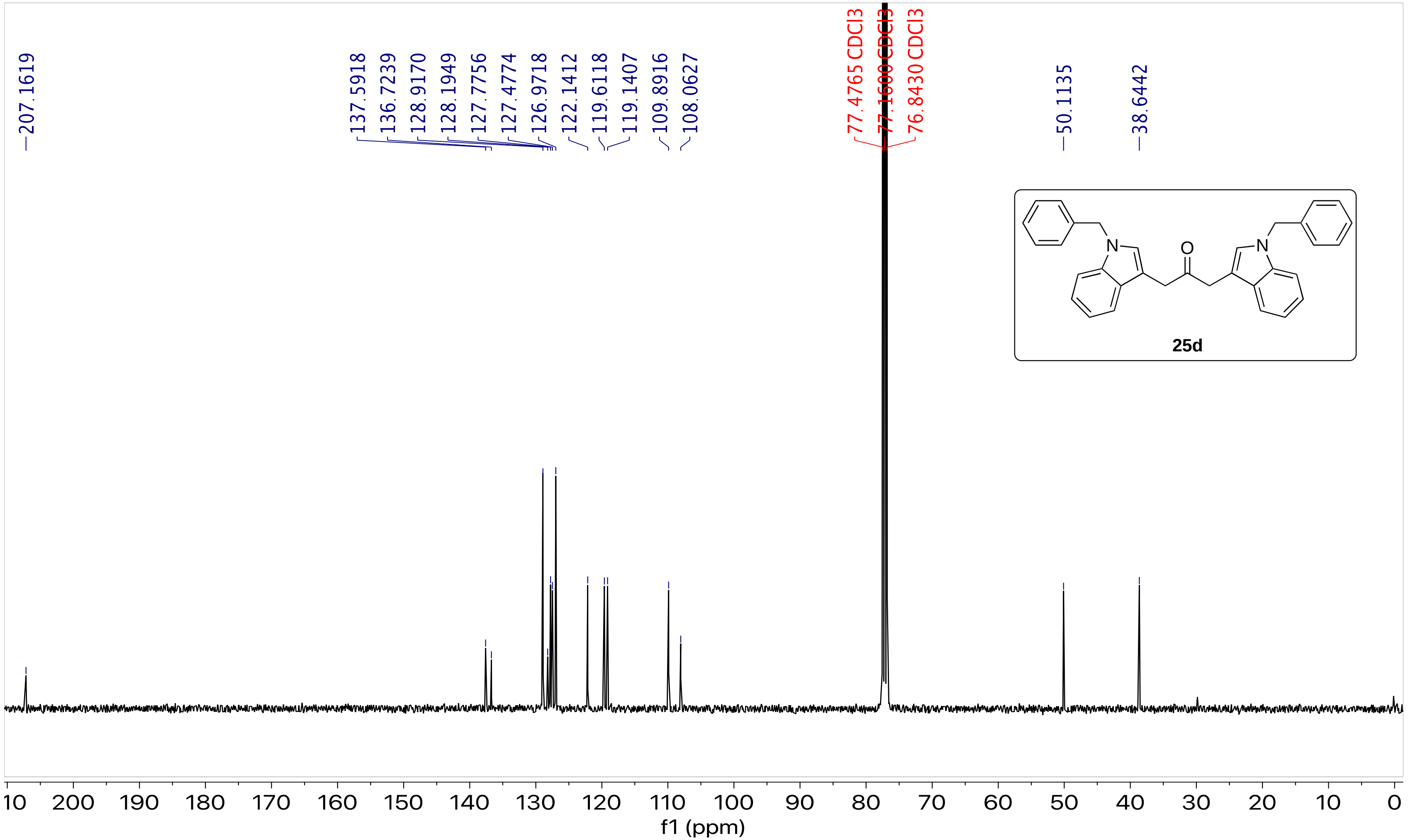

Figure S22. High-resolution mass spectrum of 2-(1-(*tert*-butoxycarbonyl)-1*H*-indol-3-yl)acetic acid (**22**)

Analysis Info

Analysis Name	D:\Data\Xiao\Nov 20 2024000009.d	Acquisition Date	2024-11-20 11:33:42 AM
Method	Xiao all 1.m	Operator	x
Sample Name	EXP-244	Instrument	compact 8255754.20059
Comment			

Acquisition Parameter

Source Type	ESI	Ion Polarity	Positive	Set Nebulizer	1.0 Bar
Focus	Not active	Set Capillary	3500 V	Set Dry Heater	181 °C
Scan Begin	50 m/z	Set End Plate Offset	-500 V	Set Dry Gas	5.0 l/min
Scan End	1500 m/z	Set Charging Voltage	2000 V	Set Divert Valve	Source
		Set Corona	0 nA	Set APCI Heater	0 °C

Meas. m/z	Ion Formula	m/z	err [ppm]
298.1057	C15H17NNaO4	298.1050	-2.4

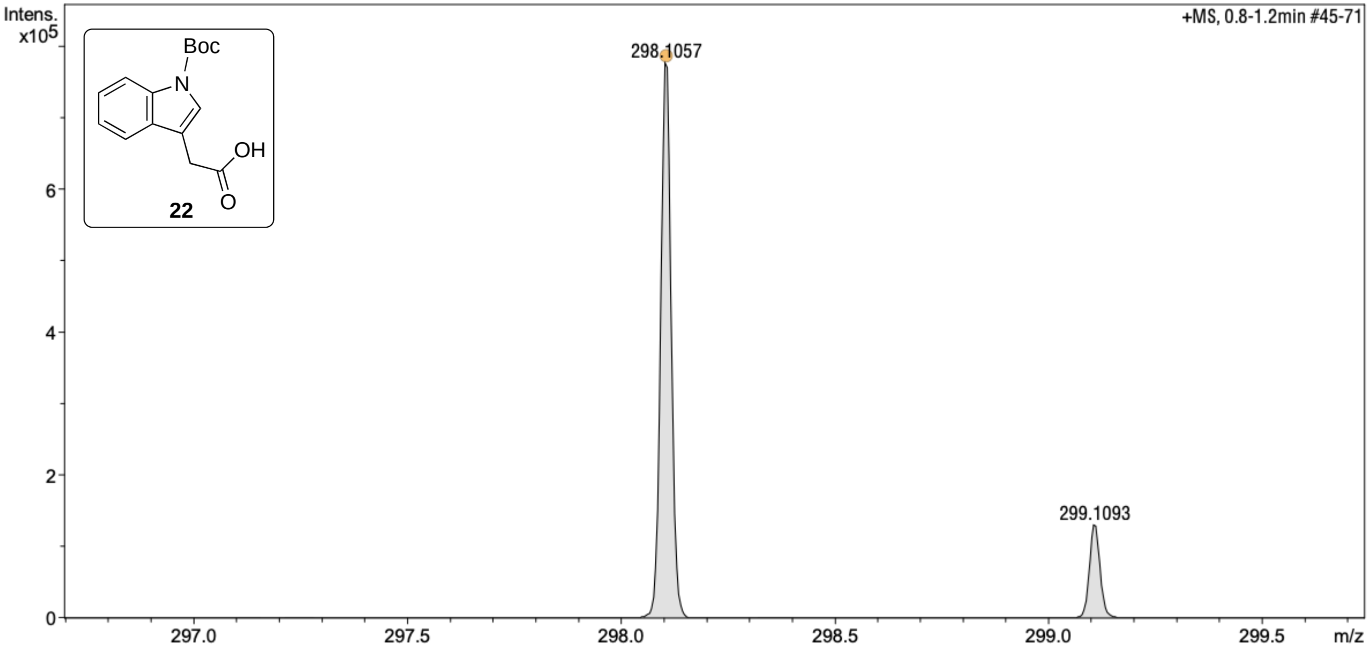

Nov 20 2024000009.d

Bruker Compass DataAnalysis 4.3 printed: 2024-11-20 11:38:29 AM by: x Page 1 of 1

Figure S23. High-resolution mass spectrum of di-*tert*-butyl 3,3'-(2-oxopropane-1,3-diyl)bis(1*H*-indole-1-carboxylate) (**23**)

Analysis Info

Analysis Name	D:\Data\Xiao\Nov 20 2024000010.d	Acquisition Date	2024-11-20 11:44:25 AM
Method	Xiao all 1.m	Operator	x
Sample Name	EXP-260	Instrument	compact 8255754.20059
Comment			

Acquisition Parameter

Source Type	ESI	Ion Polarity	Positive	Set Nebulizer	1.0 Bar
Focus	Not active	Set Capillary	3500 V	Set Dry Heater	181 °C
Scan Begin	50 m/z	Set End Plate Offset	-500 V	Set Dry Gas	5.0 l/min
Scan End	1500 m/z	Set Charging Voltage	2000 V	Set Divert Valve	Source
		Set Corona	0 nA	Set APCI Heater	0 °C

Meas. m/z	Ion Formula	m/z	err [ppm]
511.2222	C29H32N2NaO5	511.2203	-3.7

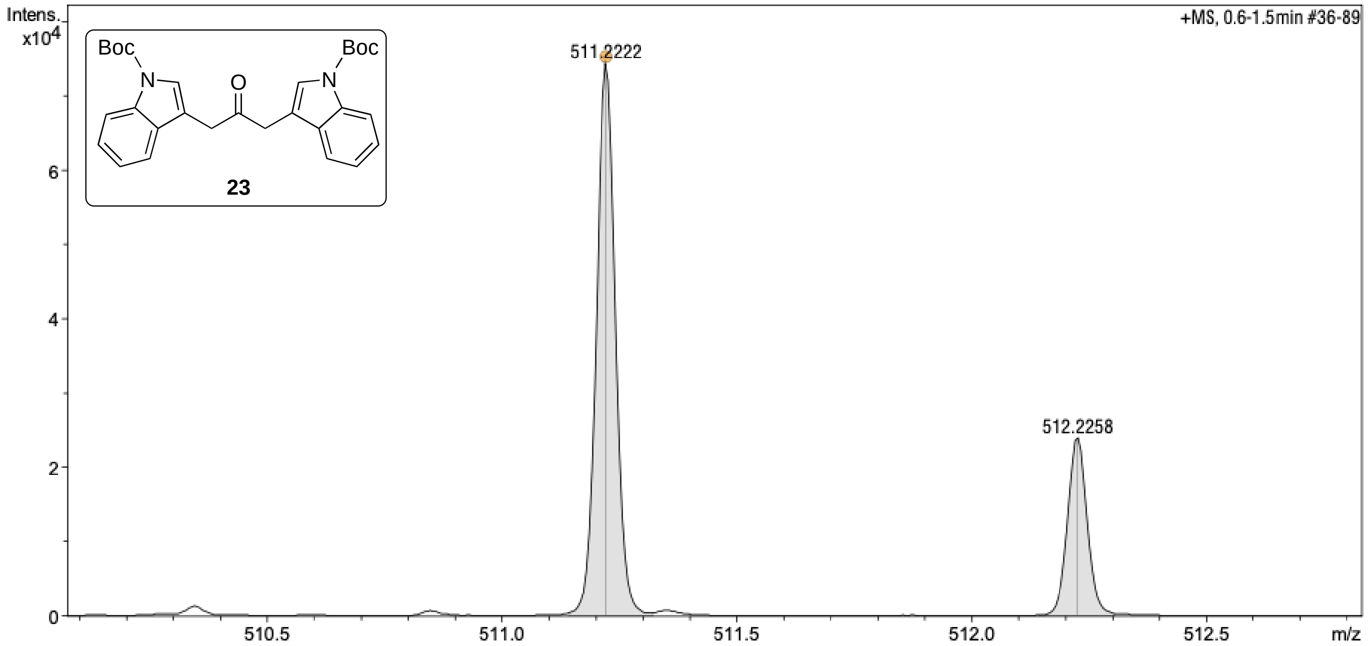

Nov 20 2024000010.d

Bruker Compass DataAnalysis 4.3 printed: 2024-11-20 11:48:48 AM by: x Page 1 of 1

Figure S24. High resolution mass spectrum of 1,3-di(1*H*-indol-3-yl)propan-2-one (**1**)

Analysis Info

Analysis Name	D:\Data\Xiao\Nov 20 2024000011.d	Acquisition Date	2024-11-20 2:06:21 PM	
Method	Xiao all 1.m	Operator	x	
Sample Name	EXP-261	Instrument	compact	8255754.20059
Comment				

Acquisition Parameter

Source Type	ESI	Ion Polarity	Positive	Set Nebulizer	1.0 Bar
Focus	Not active	Set Capillary	3500 V	Set Dry Heater	181 °C
Scan Begin	50 m/z	Set End Plate Offset	-500 V	Set Dry Gas	5.0 l/min
Scan End	1500 m/z	Set Charging Voltage	2000 V	Set Divert Valve	Source
		Set Corona	0 nA	Set APCI Heater	0 °C

Meas. m/z	Ion Formula	m/z	err [ppm]
311.1163	C19H16N2NaO	311.1155	-2.5

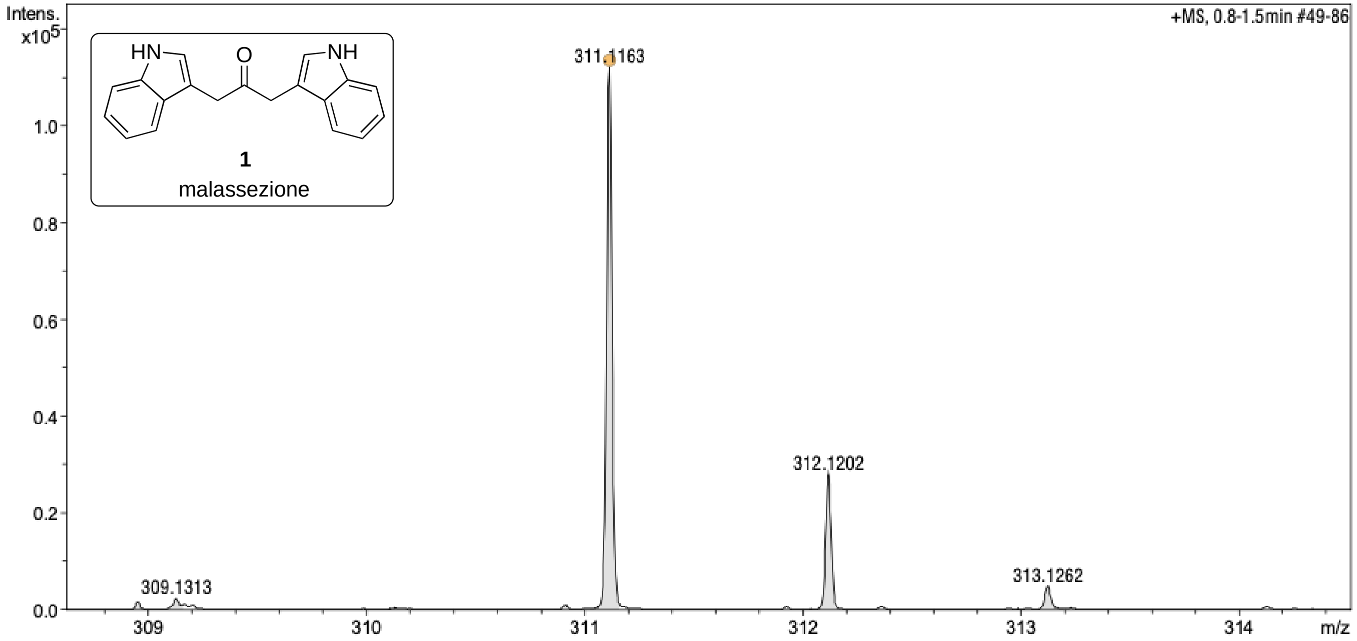

Nov 20 2024000011.d

Bruker Compass DataAnalysis 4.3 printed: 2024-11-20 2:11:11 PM by: x Page 1 of 1

Figure S25. High resolution mass spectrum of 1,3-bis(4-(benzyloxy)phenyl)propan-2-one (**25b**)

Analysis Info

Analysis Name	D:\Data\Xiao\Apl 07 2025000001.d	Acquisition Date	2025-04-07 10:54:24 AM	
Method	Xiao all 1.m	Operator	x	
Sample Name	Exp253	Instrument	compact	8255754.20059
Comment				

Acquisition Parameter

Source Type	ESI	Ion Polarity	Positive	Set Nebulizer	0.5 Bar
Focus	Not active	Set Capillary	3500 V	Set Dry Heater	180 °C
Scan Begin	50 m/z	Set End Plate Offset	-500 V	Set Dry Gas	4.0 l/min
Scan End	1500 m/z	Set Charging Voltage	2000 V	Set Divert Valve	Source
		Set Corona	0 nA	Set APCI Heater	0 °C

Meas. m/z	Ion Formula	m/z	err [ppm]
445.1777	C29H26NaO3	445.1774	-0.7

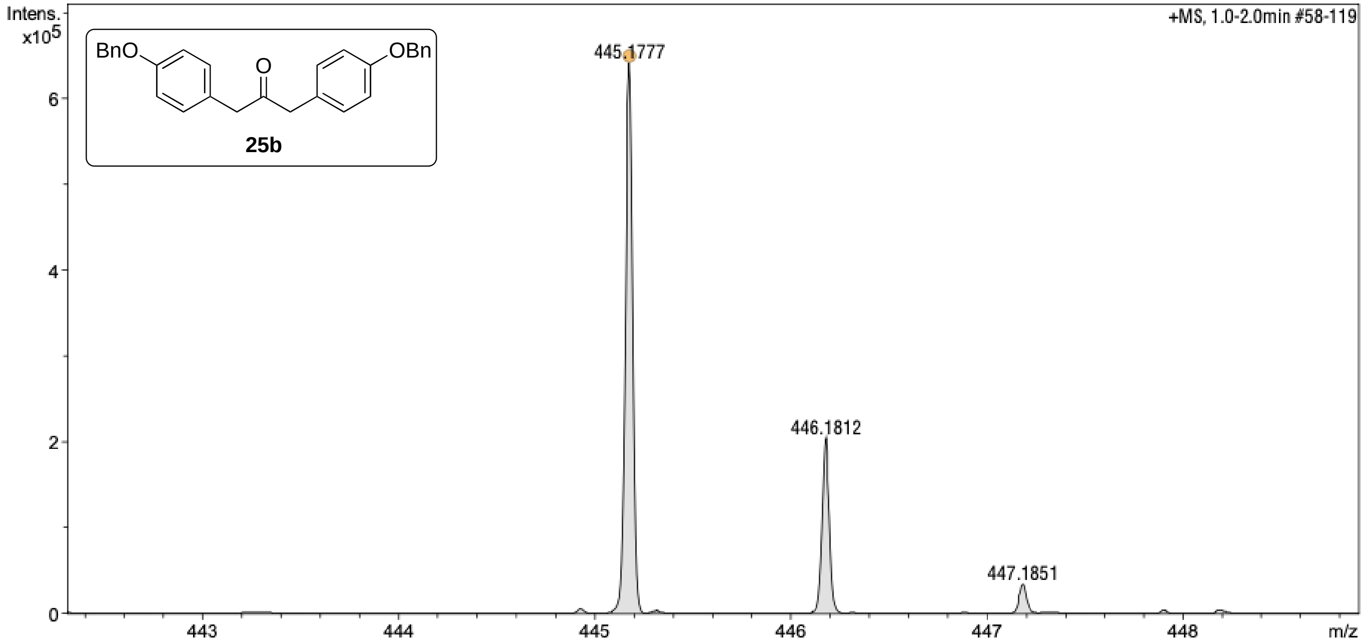

Apl 07 2025000001.d

Bruker Compass DataAnalysis 4.3 printed: 2025-04-07 10:59:49 AM by: x Page 1 of 1

Figure S26. High resolution mass spectrum of 1,3-bis(4-hydroxyphenyl)propan-2-one (**25c**)

Analysis Info

Analysis Name	D:\Data\Xiao\Apl 07 2025000008.d	Acquisition Date	2025-04-07 1:47:23 PM	
Method	Xiao all 1.m	Operator	x	
Sample Name	Exp300	Instrument	compact	8255754.20059
Comment				

Acquisition Parameter

Source Type	ESI	Ion Polarity	Positive	Set Nebulizer	0.5 Bar
Focus	Not active	Set Capillary	3500 V	Set Dry Heater	180 °C
Scan Begin	50 m/z	Set End Plate Offset	-500 V	Set Dry Gas	4.0 l/min
Scan End	1500 m/z	Set Charging Voltage	2000 V	Set Divert Valve	Source
		Set Corona	0 nA	Set APCI Heater	0 °C

Meas. m/z	Ion Formula	m/z	err [ppm]
265.0842	C15H14NaO3	265.0835	-2.5

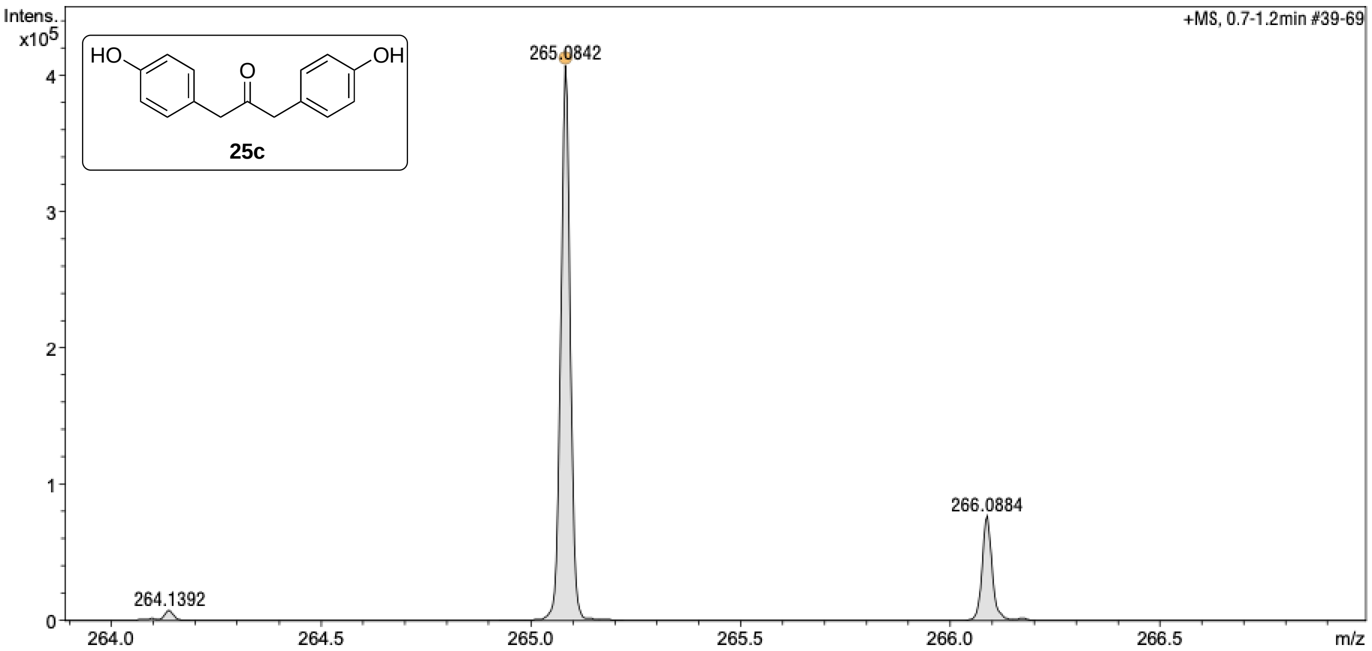

Apl 07 2025000008.d

Bruker Compass DataAnalysis 4.3 printed: 2025-04-07 1:53:27 PM by: x Page 1 of 1

Figure S27. High-resolution mass spectrum of 1,3-bis(1-benzyl-1*H*-indol-2-yl)propan-2-one (**25d**)

Analysis Info

Analysis Name	D:\Data\Xiao\Apl 07 2025000007.d	Acquisition Date	2025-04-07 1:36:40 PM	
Method	Xiao all 1.m	Operator	x	
Sample Name	Exp223	Instrument	compact	8255754.20059
Comment				

Acquisition Parameter

Source Type	ESI	Ion Polarity	Positive	Set Nebulizer	0.5 Bar
Focus	Not active	Set Capillary	3500 V	Set Dry Heater	180 °C
Scan Begin	50 m/z	Set End Plate Offset	-500 V	Set Dry Gas	4.0 l/min
Scan End	1500 m/z	Set Charging Voltage	2000 V	Set Divert Valve	Source
		Set Corona	0 nA	Set APCI Heater	0 °C

Meas. m/z	Ion Formula	m/z	err [ppm]
491.2090	C33H28N2NaO	491.2094	0.7

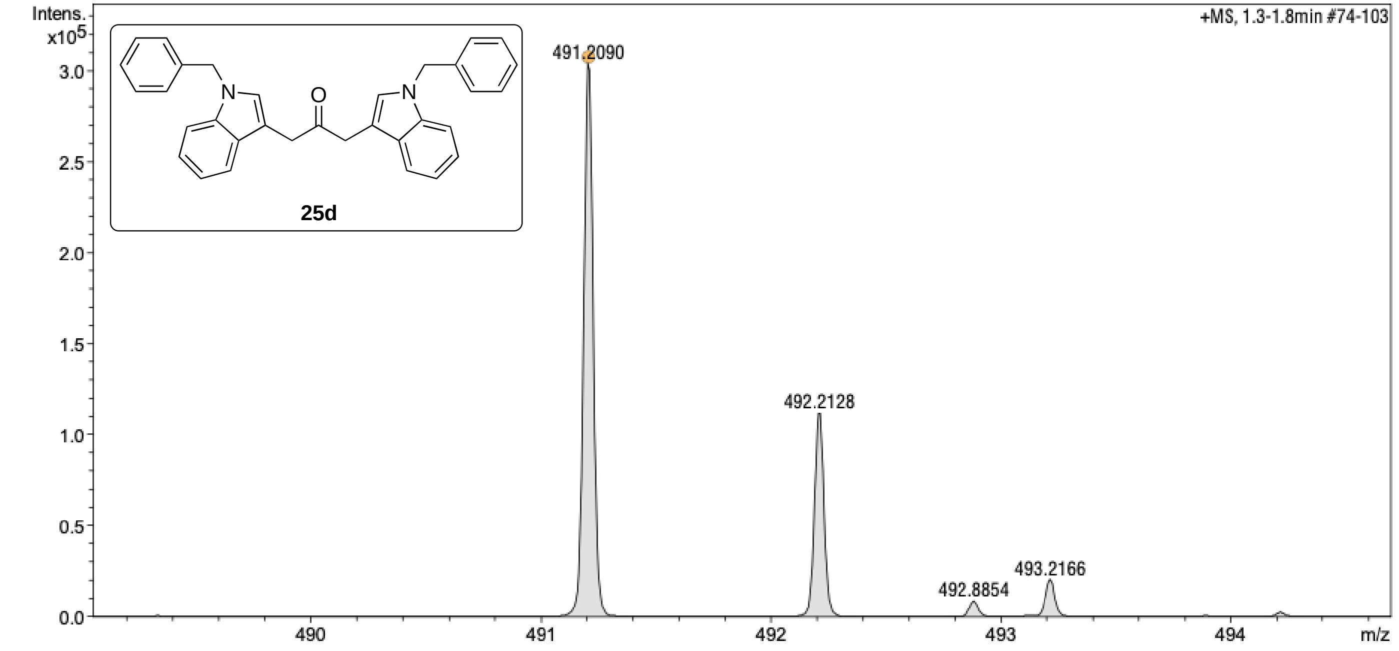

Apl 07 2025000007.d

Bruker Compass DataAnalysis 4.3 printed: 2025-04-07 1:41:26 PM by: x Page 1 of 1
